# Gender differences in the relationships between perceived individual-level occupational stress and hazardous alcohol consumption among Japanese teachers: A cross-sectional study

**DOI:** 10.1371/journal.pone.0204248

**Published:** 2018-09-20

**Authors:** Yasuhiko Deguchi, Shinichi Iwasaki, Masaru Kanchika, Tomoko Nitta, Tomoe Mitake, Yukako Nogi, Aya Kadowaki, Akihiro Niki, Koki Inoue

**Affiliations:** 1 Department of Neuropsychiatry, Osaka City University, Graduate School of Medicine, Osaka, Japan; 2 Sakura Clinic, Osaka, Japan; Zhejiang University College of Biosystems Engineering and Food Science, CHINA

## Abstract

Most teachers have a high risk of work-related stress and mental disorders. Drunken driving and hazardous alcohol consumption (HAC) among teachers are social problems. Gender differences among teachers in burnout, occupational stress, self-efficacy and job satisfaction were reported. This study aimed to clarify gender differences in the relationships between perceived individual-level occupational stress and HAC among Japanese teachers. A cross-sectional study was conducted in 2013 and a total of 723 male and 476 female teachers remained after excluding non-drinkers. Perceived individual-level occupational stress was assessed using the Generic Job Stress Questionnaire. HAC was defined as ethanol consumption greater than or equal to 280 g in 1 week for male teachers, and greater than or equal to 210 g for female teachers. Multiple logistic regression analyses were conducted. HAC was identified in 16.6% of male and 12.4% of female teachers. The average ages (± standard deviation: SD) of male and female teachers were 46.9 ± 10.9 years and 39.9 ± 12.3 years, respectively. Schoolteacher was the most common position classification among male (48.7%) and female teachers (86.3%). For those with a moderate level of stress, “social support from supervisors” was associated with HAC among males (odds ratio [OR] = 0.43, 95% confidence interval [CI] = 0.23–0.8), whereas for female teachers with a high level of stress, “variance in workload” was associated with HAC (OR = 2.09, 95% CI = 1.04–4.24), using an adjusted model. This study showed that moderate social support from supervisors was negatively related to HAC among male teachers, and high variance in workload was positively related to HAC among female teachers. Gender differences need to be considered when developing HAC prevention strategies for teachers.

## Introduction

The main reasons for alcohol consumption have been reported to be the production of positive mood states and stress-relieving effects, and alcoholic beverages are a familiar luxury good worldwide [1]. However, hazardous alcohol consumption (HAC) is reported to have associations with psychological distress such as depression, anxiety [2–5], cancer [6], hippocampal atrophy and cognitive decline [7], and reduced total brain volume [8]. HAC in the workplace can be detrimental to employees’ mental and physical health, and work productivity in the form of impaired performance, accidents, injuries, absenteeism, employee turnover, and increased healthcare costs [9–11].

Several previous studies in various countries have demonstrated that teaching is a highly challenging occupation and most teachers have a relatively high risk of work-related stress and mental disorders, such as depressive symptoms and burnout [12–17]. A Japanese nationwide business survey reported that Japanese teachers are mentally and physically distressed because of long working hours, bullying among students, truancy, classroom disruption, parental complications, curriculum revision, teacher evaluation systems, and conflicts among other teachers [18]. According to a statistical report by the Japanese Ministry of Education, Culture, Sports, Science and Technology (MEXT), two-thirds of teachers’ sick leave arises due to mental illness, and high levels of teachers’ sick leave due to mental disorders (about 5,000 per year) have been sustained for at least 9 years in Japanese public schools [19].

Teachers are under constant work-related stress, and given that occupational stress is a motive for heavy drinking, a relationship between teachers’ occupational stress and mental health problems, especially problems with HAC, is expected. The relationships between HAC and occupational stress, such as job strain [20,21] and social support [22–24], have been reported. However, as noted by Nielsen et al. (2015) [25], the effects on HAC of occupational stress other than job demand, job control, social support, and job strain are unknown, and no study has investigated the relationship between occupational stress and HAC among teachers. Previous studies reported gender differences among teachers in burnout [13,26], occupational stress [26], self-efficacy, and job satisfaction [27]. It has been reported that females progress to alcoholic intoxication more quickly than do males [28]. Moreover, females tend to have higher concentrations of alcohol in the blood due to differences in alcohol metabolism, despite consuming similar amounts of alcohol [29–31], and the possibility of gender-related differences in innate hormone profiles and genetic risk factors might be related to females’ higher vulnerability to alcohol intoxication compared to males [32]. However, gender differences in the relationships between perceived individual-level occupational stress and HAC remain unknown.

We hypothesized that gender differences exist with respect to the influence of teachers’ perceived individual-level occupational stress on HAC. This stress includes not only job demands, job control, social support, and job strain, but also role problems and variance in workload. The aims of this study were 1) to clarify gender differences in the relationship between perceived individual-level occupational stress and HAC among Japanese teachers, and 2) to clarify whether perceived individual-level occupational stress is related to HAC.

## Materials and methods

### Study design and participants

This study was based on data from a cross-sectional study of public school faculty members in a city in the Kansai region of Japan in 2013. We requested the principals of all the public schools for cooperation regarding teachers’ participation in our study. We asked the principals about the number of possible collaborators before sending out the questionnaires and we mailed self-administered, anonymous questionnaires with a return-mail envelope to 2,876 participants, including schoolteachers, principals, and vice-principals, who were attached to 60 kindergartens, 299 primary schools, 130 junior high schools, 21 high schools, and 11 special schools in the city. To protect individual privacy, we did not obtain identifiers of each participant’s school. In addition, to obtain informed consent, a letter was enclosed describing the aims and procedures of the study, particularly to assure participants that the survey was anonymous and voluntary, that no individual would be identified in analyzing or reporting the data, and that there was no penalty for choosing not to participate.

A total of 1,912 individuals who agreed with the aims of this study returned the questionnaire in the sealed envelope, providing a response rate of 66.5%. We excluded teachers having at least one missing entry in the questionnaire and male teachers working in kindergartens because of the small numbers of such individuals. The final number of respondents included in the analyses was 1,597. Some studies have identified a J-shaped relationship between alcohol consumption and mental disorders [3–5]. The J-shaped curve indicates that non-drinkers and heavy drinkers have a high risk of mental disorders; non-drinkers were excluded in this study to clarify the relationships between perceived individual-level occupational stress and HAC. After excluding 149 male and 249 female non-drinkers, a total of 723 male and 476 female teachers remained as subjects in this study.

### Ethics statement

The study design was approved by the Human Subjects Review Committee at Osaka City University (authorization number: 1409) prior to the study. All data were stored only in our database, and the employer did not have access to the data or know who had participated in the study.

### Demographic and work-related variables

Demographic variables included age and marital status (married or single). Work-related variables included the types of school the respondents worked at (kindergarten, primary school, junior high school, high school, or special needs education school) and position classification (principals, vice-principals, and schoolteachers).

### Measures of perceived individual-level occupational stress

Perceived individual-level occupational stress was assessed using the Generic Job Stress Questionnaire (GJSQ) developed by the National Institute for Occupational Safety and Health (NIOSH) [33]. The Japanese version of the GJSQ has demonstrated sufficient reliability and validity [34,35]. The “Job Content Questionnaire” based on the GJSQ can be used for a brief evaluation of some factors of occupational stress, such as quantitative workload, job control, social support (by supervisors and co-workers) [36]. Role problems are evaluated using the “Role conflict and ambiguity scale” [37]. However, in order to evaluate the variance in workload and other occupational stress by the same instrument, we chose the GJSQ because it can assess multilateral aspects of occupational stress, including stress reactions both at group and individual levels. The developers of the GJSQ permit use of its independent subscales to assess occupational stress [33]. We focused on the following 5 subscales (44 items) to assess occupational stress: quantitative workload, job control, role conflict, role ambiguity, and variance in workload, and we chose two subscales (8 items) measuring social support (by supervisors and co-workers) that function as a buffering factor, according to the results of previous studies [20–25]. The present study is based on the NIOSH job stress model [33]. For the job control and social support items, item descriptions are positively oriented, so that higher scores indicate lower stress. In contrast, all other items are negatively oriented, so that higher scores indicate greater stress.

Quantitative workload refers to the amount of work that a person has to deal with on a daily basis. Job control refers to the extent to which the individual feels that his or her tasks, workplace setting, and decisions at work are controllable. Variance in workload measures the extent to which the work tasks demand speed and concentration, and are generally difficult to handle. Role ambiguity refers to vague and unclear expectations set for employees, such that employees are uncertain of what is expected of them [38]. Role conflict refers to simultaneous contradictory expectations from work colleagues that interfere with one another and make it difficult to complete work-related tasks [38]. Social support from supervisors and co-workers measures the existence of avenues for acquiring social support during work time.

### Measures of alcohol consumption

To assess alcohol drinking frequency per week, 5 options were given: “rare or never,” “1 to 2 days,” “3 to 4 days,” “5 to 6 days,” and “every day.” To assess alcohol consumption amount per day, 5 options were given: “none,” “< 20 g,” “20 to 40 g,” “41 g to 60 g,” “> 60 g.” We evaluated the weekly alcohol consumption by combining the two aspects.

Based on WHO reports and a previous meta-analysis, HAC was defined as ethanol consumption greater than or equal to 280 g in 1 week for male teachers, and greater than or equal to 210 g for female teachers, while normal alcohol consumption (NAC) was defined as ethanol consumption of less than 280 g for male teachers and less than 210 g for female teachers [21,39]. Specifically, three groups of male teachers and six groups of female teachers were defined as exhibiting HAC. For males, the groups were “5 to 6 days” and “<60g,” “every day” and “41 g to 60 g,” and “every day” and “<60g.” For females, the groups were “every day” and “20 to 40 g,” “5 to 6 days” and “41 g to 60 g,” “every day” and “41 g to 60 g,” or “3 to 4 days” and “> 60 g,” “5 to 6 days” and “> 60 g,” or “every day” and “> 60 g”.

### Statistical analyses

Univariate logistic regression analyses were performed to estimate the odds ratios (ORs) of demographic variables (age and marital status), work-related variables (the types of school and position classification), and the seven GJSQ subscales with regard to the HAC group. The GJSQ subscales were subdivided into low, moderate, or high categories according to the tertile scores.

Subsequently, the ORs for belonging to the HAC group for male and female teachers were estimated via stepwise forward multivariate logistic analyses, adjusted for demographic variables, work-related variables, and the seven subdivided GJSQ subscales; independent variables with *p*-values less than 0.20 were selected for the stepwise forward multivariate model.

A multilevel analysis (school level) was not performed, because we could not obtain identifiers of which school each teacher belonged to. Differences were considered significant at *p* < 0.05. All statistical analyses were performed using SPSS version 21.0 software (SPSS Inc., Chicago, IL).

## Results

### Characteristics of participants

Tables 1 and 2 show the characteristics and GJSQ scores of 1199 male and female teachers. A total of 723 subjects (60.3%) were male and 476 (39.7%) were female. The prevalence of the HAC group was 16.6% in male and 12.4% in female teachers. The average age (± standard deviation: SD) of male teachers was 46.9 ± 10.9 years and the average age of female teachers was 39.9 ± 12.3 years. Primary school was the largest school category among male (44.5%) and among female teachers (38.9%). Schoolteacher was the most common position classification among male (48.7%) and female teachers (86.3%). An unpaired t-test showed a significant difference in age between male and female teachers’ alcohol consumption groups. A χ^2^ test showed a significant difference in the proportion of marital status, school category, and position classification between male and female teachers’ alcohol consumption groups.

**Table 1:** Characteristics of NAC group and HAC group among male teachers excluding non-drinkers.

	Male teachers							
		Total		NAC group		HAC group		*p-*value
		*n* = 723		*n* = 603 (83.4%)		*n* = 120 (16.6%)		
	Range	*M (SD)*	*n* (%)	*M (SD)*	*n* (%)	*M (SD)*	*n* (%)	
Age (years)		46.9 (10.9)		45.9 (11.2)		51.7 (7.3)		< 0.001
Marital status								< 0.05
Married			599 (82.8)		491 (81.4)		108 (90.0)	
Single			124 (17.2)		112 (18.6)		12 (10.0)	
School category								< 0.01
Primary school			322 (44.5)		264 (43.8)		58 (48.4)	
Junior high school			257 (35.6)		229 (37.9)		28 (23.3)	
High school			87 (12.0)		63 (10.5)		24 (20.0)	
Special needs education school			57 (7.9)		47 (7.8)		10 (8.3)	
Position classification								< 0.01
Principal			179 (24.8)		135 (22.4)		44 (36.7)	
Vice-principal			192 (26.5)		157 (26.0)		35 (29.2)	
Schoolteacher			352 (48.7)		311 (51.6)		41 (34.1)	
GJSQ scores								
Quantitative workload	11–55	41.2 (7.3)		41.4 (7.2)		40.4 (7.8)		
Job control	16–80	45.9 (11.4)		45.8 (11.2)		46.8 (12.4)		
Role conflict	8–56	30.9 (8.3)		31.1 (8.2)		30.4 (8.9)		
Role ambiguity	6–42	19.1 (5.8)		19.3 (5.7)		18.3 (6.4)		
Variance in workload	6–43	10.5 (2.9)		10.6 (2.9)		10.3 (2.9)		
Supervisors support	6–44	13.5 (4.9)		13.6 (4.8)		12.9 (5.4)		
Coworkers support	6–45	14.5 (3.8)		14.6 (3.8)		14.2 (4.0)		

NAC: normal alcohol consumption, HAC: hazardous alcohol consumption, GJSQ: generic job stress questionnaire

SD: standard deviation

**Table 2:** Characteristics of NAC group and HAC group among female teachers excluding non-drinkers.

	Female teachers							
		Total		NAC group		HAC group		*p-*value
		*n* = 476		*n* = 417 (87.6%)		*n* = 59 (12.4%)		
	Range	*M (SD)*	*n* (%)	*M (SD)*	*n* (%)	*M (SD)*	*n* (%)	
Age (years)		39.9 (12.3)		38.7 (12.1)		48.2 (9.6)		< 0.001
Marital status								
Married			210 (44.1)		171 (41.1)		39 (66.1)	< 0.001
Single			266 (55.9)		246 (58.9)		20 (33.9)	
School category								
Kindergarten			112 (23.5)		104 (24.9)		8 (13.6)	< 0.05
Primary school			185 (38.9)		162 (38.8)		23 (38.9)	
Junior high school			116 (24.4)		104 (24.9)		12 (20.4)	
High school			17 (3.6)		13 (3.2)		4 (6.8)	
Special needs education school			46 (9.6)		34 (8.2)		12 (20.3)	
Position classification								
Principal			36 (7.6)		29 (6.9)		7 (11.9)	< 0.05
Vice-principal			29 (6.1)		22 (5.3)		7 (11.9)	
Schoolteacher			411 (86.3)		366 (87.8)		45 (76.2)	
GJSQ scores								
Quantitative workload	11–55	40.9 (7.1)		40.8 (6.9)		41.7 (7.6)		
Job control	16–80	43.8 (11.2)		43.5 (11.1)		46.2 (11.3)		
Role conflict	8–56	28.9 (8.1)		28.7 (8.1)		30.1 (8.0)		
Role ambiguity	6–42	19.4 (5.4)		19.5 (5.2)		18.2 (6.3)		
Variance in workload	3–15	10.0 (3.0)		9.9 (2.9)		10.7 (3.4)		
Supervisors support	4–20	14.2 (3.9)		14.4 (3.9)		13.1 (4.3)		< 0.05
Coworkers support	4–20	16.0 (3.1)		16.1 (3.1)		15.6 (3.3)		

NAC: normal alcohol consumption, HAC: hazardous alcohol consumption, GJSQ: generic job stress questionnaire

SD: standard deviation

### Logistic regression analysis examining the association between hazardous alcohol consumption and perceived individual-level occupational stress

Table 3 shows univariate and multivariate logistic regression analysis for male teachers, using demographic variables (age and marital status), work-related variables (type of school, position classification), and each of the seven subdivided GJSQ subscales as independent variables, with the HAC group as a dependent variable; ORs were calculated. In the univariate model, HAC was associated with age, single, vice-principal or principal position, and a school type of junior high school or high school. When entering these independent variables in a stepwise multivariate logistic regression analysis, HAC was associated with age (OR = 1.07, 95% CI = 1.04–1.09) and “social support from supervisors” for participants with a moderate level of stress (OR = 0.43, 95% CI = 0.26–0.79), using an adjusted model; other factors were excluded from the model.

**Table 3:** Analysis of the risk factors for HAC by crude and stepwise multiple logistic regression analysis for male teachers.

	Male teachers					
	Univariate model			Adjusted model†		
	OR	(95% CI)	*p*	OR	(95% CI)	*p*
Age (years)	1.06	(1.04–1.09)	<0.001	1.07	(1.04–1.09)	<0.001
Marital status						
(Married)	1.00					
Single	0.49	(0.26–0.92)	0.03			
Position classification						
(School teacher)	1.00					
Vice-principal	1.69	(1.04–2.76)	0.04			
Principal	2.47	(1.54–3.96)	<0.001			
School category						
(Primary school)	1.00			1.00		
Junior high school	0.56	(0.34–0.90)	0.02	0.62	(0.39–1.02)	0.06
High school	1.73	(1.00–3.01)	0.05	1.76	(0.99–3.09)	0.05
Special needs education school	0.97	(0.46–2.03)	0.93	1.19	(0.55–2.56)	0.65
Occupational stress						
Quantitative Workload						
(Low)	1.00					
Moderate	0.88	(0.55–1.41)	0.59			
High	0.77	(0.48–1.24)	0.28			
Job Control						
(High)	1.00					
Moderate	0.91	(0.57–1.46)	0.69			
Low	0.79	(0.49–1.29)	0.35			
Role Conflict						
(Low)	1.00					
Moderate	0.95	(0.59–1.50)	0.82			
High	0.88	(0.54–1.44)	0.62			
Role Ambiguity						
(Low)	1.00					
Moderate	0.69	(0.44–1.09)	0.12			
High	0.64	(0.39–1.05)	0.08			
Variance in workload						
(Low)	1.00					
Moderate	1.19	(0.77–1.86)	0.44			
High	0.71	(0.42–1.19)	0.19			
Social Support from Supervisor						
(High)	1.00			1.00		
Moderate	0.60	(0.36–1.01)	0.05	0.46	(0.26–0.79)	<0.01
Low	1.12	(0.71–1.78)	0.62	0.68	(0.41–1.12)	0.13
Social Support from Coworkers						
(High)	1.00					
Moderate	1.16	(0.70–1.91)	0.57			
Low	1.42	(0.87–2.34)	0.17			

†: Adjusted for all listed variables.

HAC = hazardous alcohol consumption; CI = confidence interval; OR = odds ratio

Table 4 shows univariate and stepwise multivariate logistic regression analysis for female teachers, using demographic variables (age and marital status), work-related variables (type of school, position classification), and each of the seven subdivided GJSQ subscales as independent variables, with the HAC group as a dependent variable; ORs were calculated. In the univariate model, HAC was associated with age, single, principal position, a school type of special needs education school, “role ambiguity” for those with a moderate level of stress, and “variance in workload” for those with a high level of stress. When entering these independent variables in a stepwise multivariate logistic regression analysis, HAC was associated with age (OR = 1.07, 95% CI = 1.04–1.10), school category of special needs education school (OR = 3.70, 95% CI = 1.32–10.40), and “variance in workload” for those with a high level of stress (OR = 2.09, 95% CI = 1.04–4.24), using an adjusted model; other factors were excluded from the model.

**Table 4:** Analysis of risk factors for HAC by crude and stepwise multiple logistic regression analysis for female teachers.

	Female teachers					
	Univariate model			Adjusted model†		
	OR	(95% CI)	*p-*value	OR	(95% CI)	*p-*value
Age (years)	1.07	(1.04–1.10)	<0.001	1.07	(1.04–1.10)	<0.001
Marital status						
(Married)	1.00					
Single	0.36	(0.20–0.63)	<0.001			
Position classification						
(School teacher)	1.00					
Vice-principal	1.96	(0.81–4.74)	0.13			
Principal	2.59	(1.05–6.39)	0.04			
School category						
(Kindergarten)	1.00			1.00		
Primary school	1.85	(0.79–4.28)	0.15	1.15	(0.47–2.83)	0.75
Junior high school	1.50	(0.59–3.82)	0.39	1.29	(0.48–3.46)	0.61
High school	4.00	(1.06–15.15)	0.04	3.38	(0.83–13.69)	0.09
Special needs education school	4.59	(1.73–12.16)	<0.01	3.70	(1.32–10.40)	0.01
Occupational stress						
Quantitative Workload						
(Low)	1.00					
Moderate	0.86	(0.44–1.70)	0.67			
High	1.23	(0.64–2.37)	0.54			
Job Control						
(High)	1.00					
Moderate	0.62	(0.32–1.21)	0.16			
Low	0.71	(0.37–1.36)	0.29			
Role Conflict						
(Low)	1.00					
Moderate	1.69	(0.86–3.34)	0.13			
High	1.46	(0.71–2.98)	0.31			
Role Ambiguity						
(Low)	1.00					
Moderate	0.42	(0.21–0.86)	0.02			
High	0.55	(0.29–1.04)	0.07			
Variance in workload						
(Low)	1.00			1.00		
Moderate	0.88	(0.45–1.74)	0.71	0.93	(0.46–1.91)	0.85
High	2.05	(1.07–3.92)	0.03	2.09	(1.04–4.24)	0.04
Social Support from Supervisor						
(High)	1.00					
Moderate	1.63	(0.85–3.13)	0.14			
Low	0.84	(0.39–1.78)	0.64			
Social Support from Coworkers						
(High)	1.00					
Moderate	1.64	(0.78–3.48)	0.19			
Low	1.68	(0.79–3.49)	0.18			

†: Adjusted for all listed variables.

HAC = hazardous alcohol consumption; CI = confidence interval; OR = odds ratio

## Discussion

This study identified gender differences in the relationships between perceived individual-level occupational stress and HAC using the GJSQ among Japanese teachers. The results of the present study indicate a relationship between support from supervisors and HAC among male teachers, and a relationship between variance in workload and HAC among female teachers. However, there were no relationships between role problems and HAC among male and female teachers. To our knowledge, this is the first study investigating gender differences in the relationships between perceived individual-level occupational stress and HAC, and whether perceived individual-level occupational stress other than job demands, job control, social support, and job strain is related to HAC among Japanese teachers.

### The prevalence of HAC

In this study, the prevalence of HAC was 16.6% in male teachers, 12.4% in female teachers, and 14.9% in all teachers. A previous meta-analysis reported the prevalence of HAC among male and female individuals in European countries to be 1.1% to 14.5% [21]. In the Framingham study [8], the prevalence of HAC was 15.3% in males, 5.4% in females, and 10.1% in all participants. A Japanese study on males showed the prevalence of HAC to be 6.5% [23] and 15.5% [24], respectively. The prevalence of HAC among Japanese teachers might be relatively higher.

### Social support and HAC

A previous cross-sectional study among 3398 male workers reported that younger age groups tend to use alcohol to cope with job stress and older age groups tend to use alcohol for positive social enhancement [24]. Another previous cross-sectional study among 17501 male workers showed that social support increased the likelihood of heavy drinking in both younger and older age groups [23]. As such, the relationship between social support and HAC is controversial; another previous study noted that supervisors and co-workers can have various effects (positive, negative, or no effect) on alcohol consumption [22]. However, these previous studies excluded females, and research has focused on the relationship between social support and HAC only among males. In this study, moderate social support from supervisors was negatively related to HAC. However, a relationship between social support from supervisors among female teachers and HAC was not found, and there was no relationship between support from co-workers and HAC among male and female teachers. Roman et al. (1992) investigated the “enabling” of male problem drinkers in work groups and indicated that supervisors can enable heavy drinking [40]. A previous study noted that high scores for supervisor support might have resulted from men being encouraged to drink during social gatherings and meetings, while the supervisors’ tolerance for heavy drinking and resulting problems exacerbated HAC [23]. Drinking habits are common in Japan; for example, male teachers’ HAC might be related to pouring alcoholic beverages for each other while becoming acquainted with one’s subordinates or one’s supervisors at gatherings after work, and the possibility of supervisor teachers being enablers. Not too high but adequate support from supervisors might lead to the reduction of HAC among male teachers.

### Variance in workload and HAC

There has been little investigation of variance in workload as a perceived individual-level occupational stressor and few studies have focused on the relationship between variance in workload and HAC. Previous research reported that variance in workload is related to depressive symptoms among Japanese firefighters [41] and sleep-related breathing disturbance among Japanese male workers [42]. In this study, a relationship between variance in workload and HAC was not found among male teachers, and variance in workload was positively related to HAC among female teachers. The main reported reasons for alcohol consumption are that it can produce positive mood states and that it has stress-relieving effects [1]. Based on biological mechanisms, a previous study suggested that ethanol can produce positive mood states through the central activation of the CB1 (cannabinoid 1) receptors and μ/ð-opioid receptors [1]. Variance in workload might be a strong stressor to female teachers; therefore, they attempt to produce positive mood states or relieve work-related stress through alcohol consumption. This mechanism could explain why HAC in female teachers was associated with variance in workload.

### HAC and quantitative workload, job control, role conflict, and role ambiguity

A previous cross-sectional study among 1080 Brazilian bank workers (60.7% males and 39.3% females) reported that high job strain was associated with hazardous drinking [20]. A meta-analysis of the association between job strain and alcohol intake among 142,140 participants (48.9% males and 51.1% females) demonstrated that heavy drinkers (men: consumption of 280 g or more of ethanol in 1 week, women: consumption of 210 g or more of ethanol in 1 week) are more likely to report work-related stress compared to moderate drinkers [21]. However, a previous prospective study among 3642 respondents from a wide variety of occupations (40% males and 60% females) showed that perceived job demands have little direct impact on future alcohol use [25]. According to an international, large-scale survey (the OECD Teaching and Learning International Survey: TALIS 2013), teachers in Japan have the longest working hours per week (53.9 hours in Japan vs 38.3 hours in other 34 countries) [27] and working overtime is common for Japanese teachers; therefore, we expected to find a relationship between quantitative workload and HAC. However, there was no relationship between quantitative workload, job control, and HAC among male and female teachers in this study. A previous prospective study suggested that high levels of role ambiguity or role conflict have a stronger impact on alcohol use, and interventions against excessive alcohol use and problems with alcohol use in the workplace should target work factors other than quantitative demands, decision demands, control over work intensity, and decision control [25]; few studies have focused on the relationship between role problems and HAC. According to TALIS 2013, teachers in Japan spend the most time on extracurricular activities per week (7.7 hours in Japan vs. 2.1 hours in other 34 countries) and spend less time on teaching per week (17.7 hours in Japan vs. 19.3 hours in other countries) [27]. This report indicated that Japanese teachers spend substantially more time on other tasks related to their job than they spend actually teaching. We previously reported a relationship between depressive symptoms and teachers’ high levels of role conflict and role ambiguity [15] and we expected to uncover new relationships between role problems and HAC; however, no such associations were found in this study.

### Gender difference and prevention strategy for HAC among teachers

Among male teachers in this study, moderate social support from supervisors was negatively related to HAC. Among female teachers in this study, variance in workload was positively related to HAC. The effects on HAC of perceived individual-level occupational stress differ between male and female teachers. Based on the results of this study, male supervisor teachers usually consume alcohol when communicating with their subordinates or co-workers. They might be enablers without realizing, and communication by drinking might lead to teachers’ HAC. When supervisor teachers need to communicate with their subordinates or co-workers, they should identify the possibility of being enablers and avoid enabling HAC. Rather, supervisors should aim to reduce their and their subordinates’ alcohol consumption, or they should communicate in the workplace without alcohol. Additionally, male teachers themselves have to be mindful of each other’s appropriate alcohol consumption. Female teachers might consume alcohol to relieve occupational stress. From this viewpoint, reducing the variance in workload will decrease HAC among female teachers, and female teachers themselves also must be mindful of each other’s appropriate alcohol consumption at gatherings. As such, gender differences will need to be considered when developing HAC prevention strategies for Japanese teachers. Tools for personal interaction have become diverse following the introduction of IT, and social networking services (SNS) have become widespread. Any individual can easily obtain information in real time by SNS regarding meetings, and supervisors or co-workers can casually invite their subordinates to meetings via SNS. Therefore, refusing offers to drink might be difficult and the offers themselves may be a source of stress during an individual’s private time. Consequently, opportunities to participate in gatherings and alcohol consumption have increased; therefore, the number of teachers with HAC might increase in the future.

### Limitations

Several limitations of this study should be mentioned. First, the sample size was small, only Japanese teachers were surveyed, and the data were obtained from one city in Japan. Therefore, it may be difficult to generalize the findings to other regions and cultures. Second, perceived individual-level occupational stress and participants’ alcohol consumption were evaluated through self-reports; thus, the results may have been influenced by response bias, and some misclassification might exist because under-reporting of alcohol consumption is common in population-based studies [43]. Third, the moderate response rate for our survey questionnaire (66.5%) might have led to a selection bias. Fourth, a cross-sectional method was used in this study and therefore our data do not show whether the relationship between perceived individual-level occupational stress and HAC is causal, nor can the direction of any such causality be established. In future research, a cohort or longitudinal design to address the relationships between HAC and workplace factors among teachers would be beneficial. Fifth, our results may have been influenced by residual confounding from unmeasured variables, such as stress coping style, personality, and temperament [44,45]. Future study needs to consider individual factors, such as personality or temperament, as we previously reported [46]. Sixth, the representativeness of this study sample is unknown. Seventh, we evaluated the HAC just by the usual quantity and frequency of alcohol consumption. This method does not always reflect accurate drinking patterns, and future studies should take note of this point. Finally, we could not perform a multilevel analysis (school level) because of a lack of school identifiers. Therefore, we could only analyze and discuss individual level stress; future studies will need to consider the effects of school level.

## Conclusions

This study demonstrated that a moderate level of social support from supervisors was negatively related to HAC, among male teachers, variance in workload was positively related to HAC among female teachers, and role problems were not related to HAC. Gender differences must be considered when developing HAC prevention strategies for Japanese teachers.

## Supporting information

S1 File Questionnaire in Japanese. (PDF)

S2 File NIOSH-GJSQ. (PDF)

S3 File NIOSH-GJSQ score calculation. (PDF)
