# Supplementary material for: Gender differences in the relationships between perceived individual-level occupational stress and hazardous alcohol consumption among Japanese teachers: A cross-sectional study
Source: PLoS One. 2018 Sep 20;13(9):e0204248. doi: 10.1371/journal.pone.0204248 (PMC6147498; doi:10.1371/journal.pone.0204248)
Supplement: S1 File — (PDF) [file pone.0204248.s001.pdf]

〔マークの仕方〕
・記入は、HBの黒鉛筆またはシャープペンシルで、下記のマーク方法のように◯の中をていねいに塗りつぶしてください。
・訂正する場合は、プラスチック消しゴムであとが残らないように消してください。
・この用紙は、機械処理をしますので、折り曲げたり汚したりしないようご注意ください。
マーク方法 ►
次のようなマークは読み取ることができません。 うすい

数値記入例

0	1
0	0
1	1
2	2

1 性別
- 1 女性
- 2 男性

2 年齢は何歳ですか？

		歳
0	0	
1	1	
2	2	
3	3	
4	4	
5	5	
6	6	
7	7	
8	8	
9	9	

3 あなたの現在の婚姻状態は？
- 1 既婚
- 2 未婚
- 3 離婚
- 4 死別

4 家族構成
- 1 同居（親を含む）
- 2 同居（親を含まない）
- 3 一人暮らし
- 4 単身赴任

5 現在の校園に勤めて何年ですか？

		年
0	0	
1	1	
2	2	
3	3	
4	4	
5	5	
6	6	
7	7	
8	8	
9	9	

＊月数は6ヶ月以下切捨て、それ以上は切上げ

6 あなたの現在の所属校種についてお答えください。
- 1 幼稚園
- 2 小学校
- 3 中学校
- 4 高等学校
- 5 特別支援学校
- 6 その他

7 現在の役職についてお答えください。
- 1 校園長
- 2 副校長・准校長
- 3 教頭
- 4 教員(担任あり)
- 5 教員(担任なし)
- 6 教員(小、中学校の特別支援担当)
- 7 教員（副担任）

8 前記『設問7』の仕事を何年していますか?

		年
0	0	
1	1	
2	2	
3	3	
4	4	
5	5	
6	6	
7	7	
8	8	
9	9	

＊月数は6ヶ月以下切捨て、それ以上は切上げ

9 勤務時間外での学校の業務は月に何時間ありますか?
- 1 なし
- 2 10時間以内
- 3 10-20時間
- 4 21-45時間
- 5 46-80時間
- 6 81-100時間
- 7 101時間以上

10 飲酒をする頻度は週に何日ですか？
- 1 なし
- 2 1、2日
- 3 3、4日
- 4 5、6日
- 5 毎日

11 飲酒日の一日あたりの飲酒量は程度ですか？
- 1 なし
- 2 1合未満
- 3 1～2合未満
- 4 2～3合未満
- 5 3合以上

＊清酒1合（180ml）の目安
ビール中瓶1本（約500ml）
焼酎35度（80ml）
ウイスキーダブル1杯（60ml）
ワイン2杯（240ml）

12 睡眠の問題で当てはまるものをお答えください。（複数回答可）
- 1 なし
- 2 寝付きが悪い
- 3 朝早く目が覚める
- 4 夜中に覚醒して眠れない
- 5 熟睡できない
- 6 日中の眠気がある

13 平均睡眠時間は？

		時間
0	0	
1	1	
2	2	
3	3	
4	4	
5	5	
6	6	
7	7	
8	8	
9	9	

14 次の中で知っているまたは利用したことのあるものをお答えください。

	知っている	利用したことがある
教員、校園長復職支援事業	1	2
カウンセリングルーム	1	2
心の悩み電話相談	1	2
メンタルヘルス電話相談	1	2
こころ相談ネット	1	2
心の健康問題に関する相談	1	2

あなたの職場でメンタル不調を訴えられている方（休職者、通院者を含む）はおられますか。いましたら、どのような方でしょうか。
① いない
② 1人
③ 2人
④ 3人以上
⑤ 私自身

（②～④の場合は右の表へ）

（左記に２、３、４と回答した人のみ）不調者がでたことにより変化がありましたか？	かなり減少（悪化）	少し減少（悪化）	かわらない	少し増加（改善）	かなり増加（改善）
1. 職場内でメンタルヘルス不調者の数が	①	②	③	④	⑤
2. 職場内で休みをとる人が	①	②	③	④	⑤
3. 職場内の雰囲気	①	②	③	④	⑤
4. 職場内の仕事量	①	②	③	④	⑤
5. 職場内での業務効率	①	②	③	④	⑤
6. メンタルヘルスについて話し合う機会	①	②	③	④	⑤

あなたのしている仕事について考えて下さい。

1. もしあなたが今の仕事をもう一度やるかどうか決めなければならないとしたらどうしますか？わかる範囲でお答え下さい。
   ① ためらいなく同じ仕事につくと決める　② 他の仕事についても考える　③ この仕事には絶対につかないと決める
2. もし今あなたが仕事を何でも自由に選べるとしたら、どうしますか？
   ① 同じ仕事につく　② 違う仕事につく　③ 働きたいとは思わない
3. もし、あなたの友人があなたのような仕事をしたいと話したら、あなたはその人に何と言うでしょうか？
   ① 強く勧める　② 勧めるかどうか迷う　③ 止めるように助言する
4. 全体として、自分の仕事にどのくらい満足していると言えますか？
   ① 非常に満足している　② いくらか満足している　③ あまり満足していない　④ 満足していない

現在あなたが仕事上でどのくらい影響力(裁量権)があるかをお尋ねします。<br>影響力とは、他の人の仕事を指示する権限や、自分の仕事を決める自由がどの程度あるかを意味します。次の各項目に対してどのくらい影響力があるかお答え下さい。	ほとんどない	あまりない	まあまあ	たくさん	非常にたくさん
1. 自分の仕事の種類への影響力	①	②	③	④	⑤
2. 自分の仕事に必要な消耗品や備品を手に入れることへの影響力	①	②	③	④	⑤
3. 自分の仕事の順序への影響力	①	②	③	④	⑤
4. 自分の仕事の量への影響力	①	②	③	④	⑤
5. 自分の仕事のペース（どのいくらい速くあるいはゆっくり働くか）への影響力	①	②	③	④	⑤
6. 自分の仕事の質への影響力	①	②	③	④	⑤
7. 自分の作業場所での物の配置や飾りつけへの影響力	①	②	③	④	⑤
8. あなたの職場で誰がどの作業をするかの決定への影響力	①	②	③	④	⑤
9. 自分の勤務時間または勤務スケジュールへの影響力	①	②	③	④	⑤
10. あなたの職場としていつまでに仕事をするかの決定への影響力	①	②	③	④	⑤
11. あなたの職場での仕事の方針、手順、出来高への影響力	①	②	③	④	⑤
12. 自分の仕事に必要な材料を手に入れることへの影響力	①	②	③	④	⑤
13. あなたの職場の従業員の教育・訓練への影響力	①	②	③	④	⑤
14. あなたの職場の机・いすや調度品やその他の機器を置く場所への影響力	①	②	③	④	⑤
15. 仕事を先にすすめて勤務時間中に短い休憩がとれる	①	②	③	④	⑤
16. 全体として、仕事や仕事に関連することへの影響力	①	②	③	④	⑤

次の項目はあなたの仕事のいろいろな側面に関するものです。あなたの仕事ではそれぞれがどの程度あるか数字でお答え下さい。	ほとんどない	少し	多少	かなり	非常に
1. 仕事の負荷がどのくらいゆるやかになることがありますか？	①	②	③	④	⑤
2. 集中して考える余裕はどのくらいありますか？	①	②	③	④	⑤
3. 仕事の量はどのくらいありますか？	①	②	③	④	⑤
4. あなたはどのくらいの量の仕事をすることを期待されていますか？	①	②	③	④	⑤
5. 仕事をする時間的余裕はどのくらいありますか？	①	②	③	④	⑤
6. いくつくらいの仕事（プロジェクト、割当、作業）を抱えていますか？	①	②	③	④	⑤
7. きつい仕事をしている合間に、一時的に仕事がなくなることはどのくらいありますか？	①	②	③	④	⑤
8. 他の人の将来に対してどのくらい責任がありますか？	①	②	③	④	⑤
9. 他の人の仕事上の安全に対してどのくらい責任がありますか？	①	②	③	④	⑤
10. 他の人の労働意欲（モラール）に対してどのくらい責任がありますか？	①	②	③	④	⑤
11. 他の人の福祉や生活に対してどのくらい責任がありますか？	①	②	③	④	⑤

次のようなことがあなたの仕事でどのくらいの頻度で起きるかをお答え下さい。	ほとんどない	たまに	ときどき	しばしば	よくある
1. 非常に早く働かなければならないこと	①	②	③	④	⑤
2. とても一生懸命働かなければならないこと	①	②	③	④	⑤
3. 時間がなくて仕事を処理しきれないこと	①	②	③	④	⑤
4. 非常にたくさんの仕事をしなければならなこと	①	②	③	④	⑤
5. 仕事の負荷が著しく増えること	①	②	③	④	⑤
6. 仕事に必要な集中度が著しく増えること	①	②	③	④	⑤
7. ものごとを考えるスピードが著しく速くなること	①	②	③	④	⑤
8. 学校で学んだ技能や知識を仕事で使うこと	①	②	③	④	⑤
9. 自分の得意なことをする機会	①	②	③	④	⑤
10. 以前の経験や教育・訓練で得た技能を使えること	①	②	③	④	⑤

下記の問いに右の基準でお答えください。		非常に	多少	少し	全くなし	そういう人はいない
● 次の人たちはあなたの仕事が楽にできるように、どのくらい配慮や手助けをしてくれますか？	1. 直属の上司	①	②	③	④	⑤
	2. 職場の同僚	①	②	③	④	⑤
	3. 配偶者、友達、親族	①	②	③	④	⑤
● 次の人たちとどのくらい気軽に話ができますか？	1. 直属の上司	①	②	③	④	⑤
	2. 職場の同僚	①	②	③	④	⑤
	3. 配偶者、友達、親族	①	②	③	④	⑤
● 仕事で困ったことが起きた場合、次の人たちはどのくらい頼りになりますか？	1. 直属の上司	①	②	③	④	⑤
	2. 職場の同僚	①	②	③	④	⑤
	3. 配偶者、友達、親族	①	②	③	④	⑤
● 次の人たちは、あなたの個人的な問題を相談したら、どのくらい聞いてくれますか？	1. 直属の上司	①	②	③	④	⑤
	2. 職場の同僚	①	②	③	④	⑤
	3. 配偶者、友達、親族	①	②	③	④	⑤

あなたの職場（仕事または業務のグループ）の状況について次の質問にお答え下さい。	全く違う	少し違う	どちらともいえない	だいたいあてはまる	大いにあてはまる
1. 私のグループは調和がとれている。	①	②	③	④	⑤
2. 私のグループでは誰が何をするべきかでよく口論になる。	①	②	③	④	⑤
3. 私のグループのメンバーの間で意見の違いがある。	①	②	③	④	⑤
4. 私のグループでは意見の衝突がある。	①	②	③	④	⑤
5. 私のグループのメンバーはお互いの意見を支持している。	①	②	③	④	⑤
6. 私のグループでは小グループどうしが衝突している。	①	②	③	④	⑤
7. 私のグループのメンバーの間には友好的な雰囲気がある。	①	②	③	④	⑤
8. 私のグループのメンバーの間には、仲間意識がある。	①	②	③	④	⑤
9. 私のグループと他のグループとの間に対立がある。	①	②	③	④	⑤
10. 私のグループと他のグループとの間で意見が一致している。	①	②	③	④	⑤
11. 私のグループの作業の達成に必要な情報を他のグループが知らせてくれない。	①	②	③	④	⑤
12. 全体の目標を達成するために私のグループと他のグループは協調している。	①	②	③	④	⑤
13. 私のグループと他のグループは互いの助け合いがない。	①	②	③	④	⑤
14. 私のグループと他のグループとの間には協力関係がある。	①	②	③	④	⑤
15. 私のグループと他のグループとはうまが合わない。	①	②	③	④	⑤
16. 他のグループが私のグループに対して問題を引き起こす。	①	②	③	④	⑤

人はストレスにより気持ちがおちこむことがあります。<br>この１週間に次にあげた状態をどのくらい経験しましたか。<br>該当する回答の数字をマークしてください。	ない	ときどきあった	しばしばあった	いつもあった
1. 気分が沈んでゆううつだ	①	②	③	④
2. 些細なことで泣いたり、泣きたくなる	①	②	③	④
3. 夜よく眠れない	①	②	③	④
4. 最近やせてきた	①	②	③	④
5. 便秘している	①	②	③	④
6. 胸がドキドキする	①	②	③	④
7. 何となく疲れやすい	①	②	③	④
8. 落ちつかず、じっとしていられない	①	②	③	④
9. いつもよりイライラする	①	②	③	④
10. 自分が死んだ方が、他の人は楽に暮らせると思う	①	②	③	④
11. 朝方一番気分がいい	①	②	③	④
12. 食欲はふつうにある	①	②	③	④
13. 異性の友達とつき合ってみたい	①	②	③	④
14. 気持ちはいつもさっぱりしている	①	②	③	④
15. いつもと変わりなく仕事ができる	①	②	③	④
16. 将来に希望（楽しみ）がある	①	②	③	④
17. 迷わずに物事を決めることができる	①	②	③	④
18. 役に立つ人間だと思う	①	②	③	④
19. 今の生活は充実していると思う	①	②	③	④
20. 今の生活に満足している	①	②	③	④

次の文章はあなたの仕事にどのくらいあてはまりますか？<br>右の基準でお答え下さい。	全く違う	かなり違う	どちらかといえば違う	どちらともいえない	どちらかといえばあてはまる	だいたいあてはまる	大いにあてはまる
1. 自分にどのくらいの権限があるのかはっきりしている。	①	②	③	④	⑤	⑥	⑦
2. 自分の仕事には、計画された明確な目標や目的がある。	①	②	③	④	⑤	⑥	⑦
3. 自分がこうするべきだと思う方法とは異なったやり方で仕事をしなければならない。	①	②	③	④	⑤	⑥	⑦
4. 自分の仕事の時間を適切に配分していると思う。	①	②	③	④	⑤	⑥	⑦
5. 仕事をするのに必要な援助もないまま仕事を割り当てられる。	①	②	③	④	⑤	⑥	⑦
6. 自分の責任が何であるかわかっている。	①	②	③	④	⑤	⑥	⑦
7. 割り当てられた仕事をするために規則や方針を曲げたり破ったりしなければならない。	①	②	③	④	⑤	⑥	⑦
8. 全く違うやり方で働いている複数のグループと仕事をする。	①	②	③	④	⑤	⑥	⑦
9. 自分に何を期待されているか正確にわかっている。	①	②	③	④	⑤	⑥	⑦
10. 複数の人からお互いに矛盾したことを要求される。	①	②	③	④	⑤	⑥	⑦
11. ある人には受け入れられるが他の人には受け入れられないことになりがちな仕事をしている。	①	②	③	④	⑤	⑥	⑦
12. 十分な人員・機器や材料もないまま仕事を割り当てられる。	①	②	③	④	⑤	⑥	⑦
13. 自分の仕事で何をするべきかについてはっきり説明がされている。	①	②	③	④	⑤	⑥	⑦
14. する必要のないしごとをしている。	①	②	③	④	⑤	⑥	⑦

感情表現についての次の質問にお答え下さい。	まったくあてはまらない	あまりあてはまらない	どちらともいえない	だいたいあてはまる	非常によくあてはまる
1. 意見が対立したときは、議論しないと気がすまない	①	②	③	④	⑤
2. どんな場合でも、暴力に正当な理由があるとは思えない	①	②	③	④	⑤
3. 誰かに不愉快なことをされたら、不愉快だとはっきり言う	①	②	③	④	⑤
4. ちょっとした言い合いでも、声が大きくなる	①	②	③	④	⑤
5. 相手が先に手を出したとしても、やり返さない	①	②	③	④	⑤
6. かっとなることを抑えるのが難しいときがある	①	②	③	④	⑤
7. 陰で人から笑われているように思うことがある	①	②	③	④	⑤
8. ばかにされると、すぐ頭に血がのぼる	①	②	③	④	⑤
9. 友達の意見に賛成できないときには、はっきり言う	①	②	③	④	⑤
10. 私を苦しめようと思っている人はいない	①	②	③	④	⑤
11. いらいらしていると、すぐ顔に出る	①	②	③	④	⑤
12. でしゃばる人がいても、たしなめることができない	①	②	③	④	⑤
13. たいした理由もなくかっとなることがある	①	②	③	④	⑤
14. 挑発されたら、相手をなぐりたくなるかもしれない	①	②	③	④	⑤
15. 私を嫌っている人は結構いると思う	①	②	③	④	⑤
16. 人とよく意見が対立する	①	②	③	④	⑤
17. 人をなぐりたいという気持ちになることがある	①	②	③	④	⑤
18. 人からばかにされたり、意地悪されたと感じたことはほとんどない	①	②	③	④	⑤
19. 権利を守るためには暴力もやむを得ないと思う	①	②	③	④	⑤
20. 嫌いな人に出会うことが多い	①	②	③	④	⑤
21. なぐられたら、なぐり返すと思う	①	②	③	④	⑤
22. 自分の権利は遠慮しないで主張する	①	②	③	④	⑤
23. 友人の中には、私のことを陰であれこれ言っている人がいるかもしれない	①	②	③	④	⑤
24. かっとなって、物を壊したくなることがある	①	②	③	④	⑤
