# Supplementary material for: Gender differences in the relationships between perceived individual-level occupational stress and hazardous alcohol consumption among Japanese teachers: A cross-sectional study
Source: PLoS One. 2018 Sep 20;13(9):e0204248. doi: 10.1371/journal.pone.0204248 (PMC6147498; doi:10.1371/journal.pone.0204248)
Supplement: S2 File — (PDF) [file pone.0204248.s002.pdf]

NIOSH Generic Job Stress Questionnaire

National Institute for Occupational Safety and Health
Division of Applied Research and Technology
Organizational Science and Human Factors Branch
Cincinnati, OH 45226
(513) 533-8165

QUESTIONNAIRE ADMINISTRATION – PART 1

FORM NUMBER: 99, FORM REVISION: 01

STUDY ID:

There are 22 form modules included in this questionnaire. You are encouraged to use all of these forms in your survey. A complete list of the full set of forms follows. Please put a one in the appropriate box for those modules which you intend to administer in your survey.

form	rev	Title	Blank=No, 1 = Yes
17	02	Background Information**	________________
05	01	Conflict at Work	________________
06	01	Control Scale	________________
07	01	Employment Opportunities	________________
14	01	General Health	________________
01	02	General Job Information	________________
15	01	Health Condition	________________
13	01	How do you feel about yourself	________________
09	02	Job Requirements	________________
18	01	Job Satisfaction	________________
11	01	Mental Demands	________________
12	01	Non Work Activities	________________
16	02	Other Health Information	________________
03	01	Physical Environment	________________
20	01	Problems at Work	________________
08	01	Social Support	________________
02	01	Work Hazards	________________
22	01	Work Limitations	________________
10	01	Workload and Responsibility	________________
04	01	Your Job	________________
21	01	Your Job Future	________________

** Note: Form 17, **BACKGROUND INFORMATION**, should always be the first form of every packet of forms distributed to a respondent.

QUESTIONNAIRE ADMINISTRATION – PART 2

FORM NUMBER: 98, FORM REVISION: 01

STUDY ID:

If the survey is being conducted in more than one location, please complete separate **QUESTIONNAIRE ADMINISTRATION** forms for each location (Form Numbers: 98 & 99)

Date Sent:
Date Administered:

Principle Investigator
Last Name
First Name
Title
Organization
Address
Address
City
State
Zip Code
Country
Telephone Number

Location of Survey
Description
Address
Address 2
City
State
Zip Code
Country

Number of People Receiving the Form

Please add any additional information about this questionnaire that you believe is important.

Comment1:

Comment2:

Comment3:

FACTOR INFORMATION

FORM NUMBER: 96, REVISION: 01

STUDY ID:

Factor	Rev	Factor Name	Form / Revision	Factor Definition	Alpha	Study	Type
01	01	Physical Environment Evaluation	03/01	3, 4, 6, 7, 8			0
01	01	Reverse Physical Environment Evaluation		1, 2, 5, 9, 10			0
02	01	Role Conflict	04/01	3, 5, 7, 8, 10-12, 14	0.82	Nurse	0
03	01	Role Ambiguity	04/01				0
03	01	Reverse Role Ambiguity		1, 2, 4, 6, 9, 13			
04	01	Intragroup Conflict	05/01	1, 2-4, 5, 6, 7, 8	0.86	Nurse	0
05	01	Intergroup Conflict	05/01	9, 10, 11, 12, 13, 14, 15, 16	0.85	Nurse	0
06	01	Job Future Ambiguity	21/01				0
06	01	Reverse Job Future Ambiguity		1-4	0.65	Nurse	0
07	01	Perceived Control	06/01	1-16	0.90	Nurse	0
23	00	Task Control	06/01	1, 3, 4, 5, 6, 15, 16	0.85		1
24	00	Decision Control	06/01	8, 10, 11, 13	0.74		1
25	00	Physical Environment Control	06/01	7, 14	0.79		1
26	00	Resource Control	06/01	2, 12	0.82		1
08	01	Lack of Alternate Opportunity	07/01	1-3	0.80		0
09	01	Social Support from Spr	08/01	1, 4, 7, 10	0.88	0.87 Postal	0
10	01	Social Support from Cwrk	08/01	2, 5, 8, 11	0.84	0.85 Postal	0
11	01	Social Support from Family	08/01	3, 6, 9, 12	0.85	0.76 Postal	0
12	01a	Quantitative Workload	09/02	1-4			0
12	01b	Quantitative Workload	10/01	3, 4, 6	0.85	Nurse	0
12	01b	Reverse Quantitative Workload		1, 2, 5			0
13	01	Variance in Workload	09/02	5-7	0.86	Nurse	0
14	01	Responsibility for People	10/01	8-11	0.62	Nurse	0
15	01	Reverse Skill Underutilization	10/01	8, 9, 10	0.73	Nurse	0
16	01	Mental Demands	11/01	4, 5	0.75	0.71 Postal	0
17	01	Non-work Activities	12/01	1-7			0
19	01	Self-Esteem	13/01	4, 5, 8, 10	0.85	Nurse	0
19	01	Reverse Self-Esteem		1, 2, 3, 6, 7, 9			0
20	01	Somatic Complaints	14/01	1-17	0.87	Nurse	0
21	01	Reverse Job Satisfaction	18/01	1-4	0.83	Nurse	0
22	01	Depression	16/02	6-8, 10-12, 14-16, 18-20, 22-25			0
22	01	Reverse Depression		9, 13, 17, 21			0

Type 0 = Subjective Assessment

Type 1 = Principle Component with oblique Rotation

BACKGROUND INFORMATION

FORM NUMBER: 17, FORM REVISION: 02

STUDY ID:

We want to know about your work environment and how it affects you. This information is not available anywhere else. Your answers on the enclosed forms are needed.

DO NOT PUT YOUR NAME ON ANY OF THE FORMS PROVIDED. Your answers are to remain anonymous. The information which you provide will be combined with other answers only in statistical summaries.

Thank you for your cooperation and support.

1. What is your Gender?
   1 = Female
   2 = Male

2. How old were you on your last birthday? (in years)

3. What is your marital status?
   1 = Married
   2 = Single, Never Married
   3 = Single, Divorced
   4 = Single, Widowed

If you have children living at home, how many are in each of the following age groups:

4. Less than 4 years old

5. 4 Through 12 years old

6. 13 through 18 years old

7. 19 and over

CONFLICT AT WORK

FORM NUMBER: 05, FORM REVISION: 01

STUDY ID:

Please answer the following questions about your work situation. Please enter a number in the space provided at the end of each statement.

1. There is harmony within my group.
   1 Strongly Disagree
   2 Moderately Disagree
   3 Neither Agree nor Disagree
   4 Moderately Agree
   5 Strongly Agree

2. In our group, we have lots of bickering over who should do what job.
   1 Strongly Disagree
   2 Moderately Disagree
   3 Neither Agree nor Disagree
   4 Moderately Agree
   5 Strongly Agree

3. There is difference of opinion among the members of my group.
   1 Strongly Disagree
   2 Moderately Disagree
   3 Neither Agree nor Disagree
   4 Moderately Agree
   5 Strongly Agree

4. There is dissension in my group.
   1 Strongly Disagree
   2 Moderately Disagree
   3 Neither Agree nor Disagree
   4 Moderately Agree
   5 Strongly Agree

5. The members of my group are supportive of each other's ideas.
   1 Strongly Disagree
   2 Moderately Disagree
   3 Neither Agree nor Disagree
   4 Moderately Agree
   5 Strongly Agree

6. There are clashes between subgroups within my group.
   1 Strongly Disagree
   2 Moderately Disagree
   3 Neither Agree nor Disagree
   4 Moderately Agree
   5 Strongly Agree

7. There is friendliness among the members of my group.
   1 Strongly Disagree
   2 Moderately Disagree
   3 Neither Agree nor Disagree
   4 Moderately Agree
   5 Strongly Agree

8. There is "we" feeling among members of my group.
   1 Strongly Disagree
   2 Moderately Disagree
   3 Neither Agree nor Disagree
   4 Moderately Agree
   5 Strongly Agree

9. There are disputes between my group and other groups.
   1 Strongly Disagree
   2 Moderately Disagree
   3 Neither Agree nor Disagree
   4 Moderately Agree
   5 Strongly Agree

10. There is agreement between my group and other groups.
   1 Strongly Disagree
   2 Moderately Disagree
   3 Neither Agree nor Disagree
   4 Moderately Agree
   5 Strongly Agree

11. Other groups withhold information necessary for the attainment of our group tasks.
   1 Strongly Disagree
   2 Moderately Disagree
   3 Neither Agree nor Disagree
   4 Moderately Agree
   5 Strongly Agree

12. The relationship between my group and other groups is harmonious in attaining the overall organizational goals.
   1 Strongly Disagree
   2 Moderately Disagree
   3 Neither Agree nor Disagree
   4 Moderately Agree
   5 Strongly Agree

13. There is lack of mutual assistance between my group and other groups.
   1 Strongly Disagree
   2 Moderately Disagree
   3 Neither Agree nor Disagree
   4 Moderately Agree
   5 Strongly Agree

14. There is cooperation between my group and other groups.
    1 Strongly Disagree
    2 Moderately Disagree
    3 Neither Agree nor Disagree
    4 Moderately Agree
    5 Strongly Agree

15. There are personality clashes between my group and other groups.
    1 Strongly Disagree
    2 Moderately Disagree
    3 Neither Agree nor Disagree
    4 Moderately Agree
    5 Strongly Agree

16. Other groups create problems for my group.
    1 Strongly Disagree
    2 Moderately Disagree
    3 Neither Agree nor Disagree
    4 Moderately Agree
    5 Strongly Agree

EMPLOYMENT OPPORTUNITIES

FORM NUMBER: 07, FORM REVISION: 01

STUDY ID:

The next four questions ask you to evaluate your feelings about your job in relationship to other jobs you might be able to get. Please respond to each item by placing the number of the response that best indicates your feelings about the question in the space provided at the end of each question.

1. How easy would it be for you to find a suitable job with another employer?
    - 1 Very Easy
    - 2 Quite Easy
    - 3 Fairly Easy
    - 4 Not Quite so Easy
    - 5 Not at all Easy

2. How easy would it be for you to find a job as good *as the one you now have* with another employer?
    - 1 Very Easy
    - 2 Quite Easy
    - 3 Fairly Easy
    - 4 Not Quite so Easy
    - 5 Not at all Easy

3. How would you describe the *number of available jobs*, with all types of employers, for a person with your qualifications?
    - 1 Very Easy
    - 2 Quite Easy
    - 3 Fairly Easy
    - 4 Not Quite so Easy
    - 5 Not at all Easy

4. How likely is it that you would have to move out of your local area to find a suitable job with another employer?
    - 1 Very Easy
    - 2 Quite Easy
    - 3 Fairly Easy
    - 4 Not Quite so Easy
    - 5 Not at all Easy

GENERAL HEALTH

FORM NUMBER: 14, FORM REVISION: 01

STUDY ID:

This portion of the questionnaire contains items that are related to general health. These items are not necessarily related to severe physical illness but are things that people experience in their day to day lives.

Please enter a number in the space provided at the end of each statement.

How often have you experienced any of the following during the past month?

1. Your face became hot when you were not in a hot room or exercising.
   1 Never
   2 Occasionally
   3 Sometimes
   4 Fairly Often
   5 Very Often

2. You perspired excessively when you were not in a hot room or exercising.
   1 Never
   2 Occasionally
   3 Sometimes
   4 Fairly Often
   5 Very Often

3. Your mouth became dry.
   1 Never
   2 Occasionally
   3 Sometimes
   4 Fairly Often
   5 Very Often

4. Your muscles felt tight and tense.
   1 Never
   2 Occasionally
   3 Sometimes
   4 Fairly Often
   5 Very Often

5. You were bothered by a headache.
   1 Never
   2 Occasionally
   3 Sometimes
   4 Fairly Often
   5 Very Often

6. You felt as if the blood were rushing to your head.
   1 Never
   2 Occasionally
   3 Sometimes
   4 Fairly Often
   5 Very Often

7. You felt a lump in your throat or a choked-up feeling.
    1 Never
    2 Occasionally
    3 Sometimes
    4 Fairly Often
    5 Very Often

8. Your hands trembled enough to bother you.
    1 Never
    2 Occasionally
    3 Sometimes
    4 Fairly Often
    5 Very Often

9. You were bothered by shortness of breath when you were not working hard or exercising.
    1 Never
    2 Occasionally
    3 Sometimes
    4 Fairly Often
    5 Very Often

10. You were bothered by your heart beating hard.
    1 Never
    2 Occasionally
    3 Sometimes
    4 Fairly Often
    5 Very Often

11. Your hands sweated so that you felt damp and clammy.
    1 Never
    2 Occasionally
    3 Sometimes
    4 Fairly Often
    5 Very Often

12. You had spells of dizziness.
    1 Never
    2 Occasionally
    3 Sometimes
    4 Fairly Often
    5 Very Often

13. You were bothered by having an upset stomach or stomach ache.
    1 Never
    2 Occasionally
    3 Sometimes
    4 Fairly Often
    5 Very Often

14. You were bothered by your heart beating.
    1 Never
    2 Occasionally
    3 Sometimes
    4 Fairly Often
    5 Very Often

15. You were in ill health which affected your work.
    1 Never
    2 Occasionally
    3 Sometimes
    4 Fairly Often
    5 Very Often

16. You had a loss of appetite.
    1 Never
    2 Occasionally
    3 Sometimes
    4 Fairly Often
    5 Very Often

17. You had trouble sleeping at night.
    1 Never
    2 Occasionally
    3 Sometimes
    4 Fairly Often
    5 Very Often

HEALTH CONDITIONS

FORM NUMBER: 15, FORM REVISION: 01

STUDY ID:

Within the past twelve months, has a doctor ever treated you for, or told you that you had any of the following: Please enter 1 for No or 2 for Yes.

1. Diabetes
2. Cancer
3. Hernia or rupture
4. Tuberculosis
5. Asthma
6. "High" blood pressure
7. Heart disease
8. Arthritis
9. Epilepsy
10. Glaucoma
11. Paralysis, tremor or shaking
12. Kidney or bladder trouble
13. Lung or breathing problems
14. Stroke
15. Anemia
16. Gall Bladder, liver, or pancreas trouble
17. Thyroid trouble or goiter
18. Insomnia
19. Gastritis
20. Colitis
21. Stomach ulcer
22. Alcoholism
23. Emotional problems
24. Back problems

HOW DO YOU FEEL ABOUT YOURSELF

FORM NUMBER: 13, FORM REVISION: 01

STUDY ID:

Please indicate how strongly you agree or disagree with the following statements by entering a number on the space provided at the end of each statement.

1. On the whole, I am satisfied with myself.
    1 Strongly Disagree
    2 Disagree
    3 Neither Agree nor Disagree
    4 Agree
    5 Strongly Agree

2. I feel I do not have much to be proud of.
    1 Strongly Disagree
    2 Disagree
    3 Neither Agree nor Disagree
    4 Agree
    5 Strongly Agree

3. I certainly feel useless at times.
    1 Strongly Disagree
    2 Disagree
    3 Neither Agree nor Disagree
    4 Agree
    5 Strongly Agree

4. I feel that I'm a person of worth, at least on an equal basis with others.
    1 Strongly Disagree
    2 Disagree
    3 Neither Agree nor Disagree
    4 Agree
    5 Strongly Agree

5. I feel that I have a number of good qualities.
    1 Strongly Disagree
    2 Disagree
    3 Neither Agree nor Disagree
    4 Agree
    5 Strongly Agree

6. All in all, I am inclined to feel that I am a failure.
    1 Strongly Disagree
    2 Disagree
    3 Neither Agree nor Disagree
    4 Agree
    5 Strongly Agree

7. I wish I could have more respect for myself.
   1 Strongly Disagree
   2 Disagree
   3 Neither Agree nor Disagree
   4 Agree
   5 Strongly Agree

8. I am able to do things as well as most other people.
   1 Strongly Disagree
   2 Disagree
   3 Neither Agree nor Disagree
   4 Agree
   5 Strongly Agree

9. At times I think I am no good at all.
   1 Strongly Disagree
   2 Disagree
   3 Neither Agree nor Disagree
   4 Agree
   5 Strongly Agree

10. I take a positive attitude toward myself.
   1 Strongly Disagree
   2 Disagree
   3 Neither Agree nor Disagree
   4 Agree
   5 Strongly Agree

GENERAL JOB INFORMATION

FORM NUMBER: 01, FORM REVISION: 03

STUDY ID:

Please answer the following questions.

1. How long have you worked with your present employer?
   Years:
   Months:

2. What is your current JOB TITLE?

3. JOB TITLE CODE:

4. How long have you worked in this job?
   Years:
   Months:

5. Select the most appropriate description of your JOB SITUATION:
   1 Full-time permanent employee
   2 Full-time temporary employee
   3 Part-time permanent employee
   4 Casual
   5 Other

6. Select the description that comes closest to your present WORK SHIFT:
   1 Rotating eight-hour shift
   2 Rotating twelve-hour shift
   3 Permanent day shift
   4 Permanent evening shift
   5 Permanent night shift
   6 Other

7. How long have you worked the shift you indicated above?
   Years:
   Months:

8. IF you work on a rotating shift, what ROTATION PATTERN do you follow?
   1 **Eight-Hour Shift:** DAY to EVENING to NIGHT
   2 **Eight-Hour Shift:** NIGHT to EVENING to DAY
   3 **Eight-Hour Shift:** No set pattern
   4 **Twelve-Hour Shift:** DAY to NIGHT
   5 **Twelve-Hour Shift:** NIGHT to DAY
   6 **Twelve-Hour Shift:** No set pattern

9. How many times a week do you change shifts?
   1 0 times (I don't change)
   2 2 times
   3 More than 2 times
   4 On call
   5 Standby
   6 Non-standard work week
   7 Other

10. How many hours do you normally work per week in your job?

11. How many hours overtime do you work in your job in an average week?

12. How many hours per week do you work on any other job? (Please mark "0" if no other job)

JOB REQUIREMENTS

FORM NUMBER: 09, FORM REVISION: 02.

STUDY ID:

Now we would like you to indicate how often certain things happen at your job. Please write the number for your response in the space provided at the end of each question.

1. How often does your job require you to work *very fast*?
   1 Rarely
   2 Occasionally
   3 Sometimes
   4 Fairly Often
   5 Very Often

2. How often does your job require you to work *very hard*?
   1 Rarely
   2 Occasionally
   3 Sometimes
   4 Fairly Often
   5 Very Often

3. How often does your job leave you with *little* time to get things done?
   1 Rarely
   2 Occasionally
   3 Sometimes
   4 Fairly Often
   5 Very Often

4. How often is there a *great deal* to be done?
   1 Rarely
   2 Occasionally
   3 Sometimes
   4 Fairly Often
   5 Very Often

5. How often is there a marked increase in the work load?
   1 Rarely
   2 Occasionally
   3 Sometimes
   4 Fairly Often
   5 Very Often

6. How often is there a marked increase in the amount of concentration required on your job?
   1 Rarely
   2 Occasionally
   3 Sometimes
   4 Fairly Often
   5 Very Often

7. How often is there a marked increase in *how fast* you have to think?
    1 Rarely
    2 Occasionally
    3 Sometimes
    4 Fairly Often
    5 Very Often

8. How often does your job let you use the skills and knowledge you learned in school?
    1 Rarely
    2 Occasionally
    3 Sometimes
    4 Fairly Often
    5 Very Often

9. How often are you given a change to do the things you do the best?
    1 Rarely
    2 Occasionally
    3 Sometimes
    4 Fairly Often
    5 Very Often

10. How often can you use the skills from your previous experience and training?
    1 Rarely
    2 Occasionally
    3 Sometimes
    4 Fairly Often
    5 Very Often

JOB SATISFACTION

FORM NUMBER: 18, FORM REVISION: 01

STUDY ID:

We would like you to think about the *type of work you do in your job*.

1. Knowing what you know now, if you had to decide all over again whether to take the type of job you now have, what would you decide?
    1 I would decide without hesitation to take the same job.
    2 I would have some second thoughts.
    3 I would decide definitely NOT to take this type of job.

2. If you were free right now to go into any type of job you wanted, what would your choice be?
    1 I would take the same job.
    2 I would take a different job.
    3 I would not want to work.

3. If a friend of yours told you he/she was interested in working in a job like yours, what would you tell him/her?
    1 I would strongly recommend it.
    2 I would have doubts about recommending it.
    3 I would advise against it.

4. All in all, how satisfied would you say you are with your job?
    1 I am very satisfied.
    2 I am somewhat satisfied.
    3 I am not too satisfied.
    4 I am not at all satisfied.

MENTAL DEMANDS

FORM NUMBER: 11, FORM REVISION: 01

STUDY ID:

Please indicate the degree to which you agree or disagree with the following statements about your job. Please enter the number in the space provided at the end of each statement.

1. My job requires a great deal of concentration.
   1 Strongly Agree
   2 Slightly Agree
   3 Slightly Disagree
   4 Strongly Disagree

2. My job requires me to remember many different things.
   1 Strongly Agree
   2 Slightly Agree
   3 Slightly Disagree
   4 Strongly Disagree

3. I must keep my mind on my work at all times.
   1 Strongly Agree
   2 Slightly Agree
   3 Slightly Disagree
   4 Strongly Disagree

4. I can take it easy and still get my work done.
   1 Strongly Agree
   2 Slightly Agree
   3 Slightly Disagree
   4 Strongly Disagree

5. I can let my mind wander and still do the work
   1 Strongly Agree
   2 Slightly Agree
   3 Slightly Disagree
   4 Strongly Disagree

NON-WORK ACTIVITIES

FORM NUMBER: 12, FORM REVISION: 01

STUDY ID:

Please answer the following questions.
Please enter 0 for No or 1 for Yes in the space provided at the end of each question.

1. Do you work on another job in addition to this one?

2. Do you have any children at home?

3. Do you have primary responsibility for child care duties?

4. Do you have primary responsibility for house-cleaning duties?

5. Do you have primary responsibility for the care of an elderly or disabled person on a regular basis?

6. Are you going to school and taking courses for credit toward a degree?

7. Do you belong to a voluntary or religious organization at which you spend at least 5 to 10 hours per week?

OTHER HEALTH INFORMATION

FORM NUMBER: 16, FORM REVISION: 02

STUDY ID:

On an average *day*, how many of each of the following do you smoke?

1. Cigarettes
2. Cigars
3. Pipefuls of tobacco

4. During the past *6 months*, have you had any on the job accidents?
   1 = Yes
   2 = No

5. During the past *month*, about how many days of sick leave did you take?
(Please mark a 0 if no sick days taken.)

During the past *week*, how often did you experience the following:

6. I was bothered by things that usually don't bother me.
   0 = Rarely or none of the time (less than 1 day)
   1 = Some of the time (1-2 days)
   2 = Occasionally or a moderate amount of time (3-4 days)
   3 = Most or all of the time (5-7 days)

7. I did not feel like eating; my appetite was poor.
   0 = Rarely or none of the time (less than 1 day)
   1 = Some of the time (1-2 days)
   2 = Occasionally or a moderate amount of time (3-4 days)
   3 = Most or all of the time (5-7 days)

8. I felt that I could not shake off the blues even with help from my family or friends.
   0 = Rarely or none of the time (less than 1 day)
   1 = Some of the time (1-2 days)
   2 = Occasionally or a moderate amount of time (3-4 days)
   3 = Most or all of the time (5-7 days)

9. I felt that I was just as good as other people.
   0 = Rarely or none of the time (less than 1 day)
   1 = Some of the time (1-2 days)
   2 = Occasionally or a moderate amount of time (3-4 days)
   3 = Most or all of the time (5-7 days)

10. I had trouble keeping my mind on what I was doing.
   0 = Rarely or none of the time (less than 1 day)
   1 = Some of the time (1-2 days)
   2 = Occasionally or a moderate amount of time (3-4 days)
   3 = Most or all of the time (5-7 days)

11. I felt depressed.
    0 = Rarely or none of the time (less than 1 day)
    1 = Some of the time (1-2 days)
    2 = Occasionally or a moderate amount of time (3-4 days)
    3 = Most or all of the time (5-7 days)

12. I felt that everything I did was an effort.
    0 = Rarely or none of the time (less than 1 day)
    1 = Some of the time (1-2 days)
    2 = Occasionally or a moderate amount of time (3-4 days)
    3 = Most or all of the time (5-7 days)

13. I felt hopeful about the future.
    0 = Rarely or none of the time (less than 1 day)
    1 = Some of the time (1-2 days)
    2 = Occasionally or a moderate amount of time (3-4 days)
    3 = Most or all of the time (5-7 days)

14. I thought my life had been a failure.
    0 = Rarely or none of the time (less than 1 day)
    1 = Some of the time (1-2 days)
    2 = Occasionally or a moderate amount of time (3-4 days)
    3 = Most or all of the time (5-7 days)

15. I felt fearful.
    0 = Rarely or none of the time (less than 1 day)
    1 = Some of the time (1-2 days)
    2 = Occasionally or a moderate amount of time (3-4 days)
    3 = Most or all of the time (5-7 days)

16. My sleep was restless.
    0 = Rarely or none of the time (less than 1 day)
    1 = Some of the time (1-2 days)
    2 = Occasionally or a moderate amount of time (3-4 days)
    3 = Most or all of the time (5-7 days)

17. I was happy.
    0 = Rarely or none of the time (less than 1 day)
    1 = Some of the time (1-2 days)
    2 = Occasionally or a moderate amount of time (3-4 days)
    3 = Most or all of the time (5-7 days)

18. I talked less than usual.
    0 = Rarely or none of the time (less than 1 day)
    1 = Some of the time (1-2 days)
    2 = Occasionally or a moderate amount of time (3-4 days)
    3 = Most or all of the time (5-7 days)

19. I felt lonely.
    0 = Rarely or none of the time (less than 1 day)
    1 = Some of the time (1-2 days)
    2 = Occasionally or a moderate amount of time (3-4 days)
    3 = Most or all of the time (5-7 days)

20. People were unfriendly.
    0 = Rarely or none of the time (less than 1 day)
    1 = Some of the time (1-2 days)
    2 = Occasionally or a moderate amount of time (3-4 days)
    3 = Most or all of the time (5-7 days)

21. I enjoyed life.
    0 = Rarely or none of the time (less than 1 day)
    1 = Some of the time (1-2 days)
    2 = Occasionally or a moderate amount of time (3-4 days)
    3 = Most or all of the time (5-7 days)

22. I had crying spells.
    0 = Rarely or none of the time (less than 1 day)
    1 = Some of the time (1-2 days)
    2 = Occasionally or a moderate amount of time (3-4 days)
    3 = Most or all of the time (5-7 days)

23. I felt sad.
    0 = Rarely or none of the time (less than 1 day)
    1 = Some of the time (1-2 days)
    2 = Occasionally or a moderate amount of time (3-4 days)
    3 = Most or all of the time (5-7 days)

24. I felt that people disliked me.
    0 = Rarely or none of the time (less than 1 day)
    1 = Some of the time (1-2 days)
    2 = Occasionally or a moderate amount of time (3-4 days)
    3 = Most or all of the time (5-7 days)

25. I could not get "going".
    0 = Rarely or none of the time (less than 1 day)
    1 = Some of the time (1-2 days)
    2 = Occasionally or a moderate amount of time (3-4 days)
    3 = Most or all of the time (5-7 days)

During the past month, have you experienced any of the following:

26. Cold or flu symptoms
    1 = No
    2 = Yes

27. Hay fever or allergy symptoms.
    1 = No
    2 = Yes

28. Allergic skin rash
    1 = No
    2 = Yes

29. Slow healing wounds
    1 = No
    2 = Yes

30. Cold sores or fever blisters
    1 = No
    2 = Yes

31. Arthritis symptoms (swollen or painful joints)
    1 = No
    2 = Yes

32. Other illness
    1 = No
    2 = Yes

33. Explain (if other illness)

PHYSICAL ENVIRONMENT

FORM NUMBER: 03, FORM REVISION: 01

STUDY ID:

Please indicate whether the following statements about your job are TRUE or FALSE by entering 1 for True or 2 for False in the space provided at the end of each statement.

1. The level of **NOISE** in the area(s) in which I work is usually high.
   1 = True
   2 = False

2. The level of **LIGHTING** in the area(s) in which I work is usually poor.
   1 = True
   2 = False

3. The **TEMPERATURE** of my work area(s) during the **SUMMER** is usually comfortable.
   1 = True
   2 = False

4. The **TEMPERATURE** of my work area(s) during the **WINTER** is usually comfortable.
   1 = True
   2 = False

5. The **HUMIDITY** in my work area(s) is usually either too high or too low.
   1 = True
   2 = False

6. The level of **AIR CIRCULATION** in my work area(s) is good.
   1 = True
   2 = False

7. The **AIR** in my work area(s) is clean and free of pollution.
   1 = True
   2 = False

8. In my job, I am well protected from exposure to **DANGEROUS SUBSTANCES**.
   1 = True
   2 = False

9. The overall quality of the **PHYSICAL ENVIRONMENT** where I work is poor.
   1 = True
   2 = False

10. My **WORK AREA(S)** is/are awfully crowded.
    1 = True
    2 = False

PROBLEMS AT WORK

FORM NUMBER: 20, FORM REVISION: 01

STUDY ID:

People deal with day to day problems at work in many ways. When faced with problems at work, how often do you do each of the following: Please enter a response in the space provided at the end of each statement.

1. Make a plan to solve the problems(s) and stick to it.
   1 Rarely
   2 Occasionally
   3 Sometimes
   4 Fairly Often
   5 Very Often

2. Go on as if nothing happened.
   1 Rarely
   2 Occasionally
   3 Sometimes
   4 Fairly Often
   5 Very Often

3. Feel responsible for the problem(s).
   1 Rarely
   2 Occasionally
   3 Sometimes
   4 Fairly Often
   5 Very Often

4. Daydream or wish that you could change the problem(s).
   1 Rarely
   2 Occasionally
   3 Sometimes
   4 Fairly Often
   5 Very Often

5. Talk to your boss or co-workers about the problems(s).
   1 Rarely
   2 Occasionally
   3 Sometimes
   4 Fairly Often
   5 Very Often

6. Become more involved in activities outside of work.
   1 Rarely
   2 Occasionally
   3 Sometimes
   4 Fairly Often
   5 Very Often

SOCIAL SUPPORT

FORM NUMBER: 08, FORM REVISION: 01

STUDY ID:

How much do each of these people go out of their way to do things to *make your work life easier* for you?

1. Your immediate supervisor (boss)
    1 Very Much
    2 Somewhat
    3 A Little
    4 Not At All
    5 Don't Have Any Such Person

2. Other people at work
    1 Very Much
    2 Somewhat
    3 A Little
    4 Not At All
    5 Don't Have Any Such Person

3. Your spouse, friends and relatives
    1 Very Much
    2 Somewhat
    3 A Little
    4 Not At All
    5 Don't Have Any Such Person

How easy is it to talk with each of the following people?

4. Your immediate supervisor (boss)
    1 Very Much
    2 Somewhat
    3 A Little
    4 Not At All
    5 Don't Have Any Such Person

5. Other people at work
    1 Very Much
    2 Somewhat
    3 A Little
    4 Not At All
    5 Don't Have Any Such Person

6. Your spouse, friends and relatives
    1 Very Much
    2 Somewhat
    3 A Little
    4 Not At All
    5 Don't Have Any Such Person

How much can each of these people be *relied* on when things get tough at work?

7. Your immediate supervisor (boss)
   1 Very Much
   2 Somewhat
   3 A Little
   4 Not At All
   5 Don't Have Any Such Person

8. Other people at work
   1 Very Much
   2 Somewhat
   3 A Little
   4 Not At All
   5 Don't Have Any Such Person

9. Your spouse, friends and relatives
   1 Very Much
   2 Somewhat
   3 A Little
   4 Not At All
   5 Don't Have Any Such Person

How much is each of the following *willing to listen* to your personal problems?

10. Your immediate supervisor (boss)
    1 Very Much
    2 Somewhat
    3 A Little
    4 Not At All
    5 Don't Have Any Such Person

11. Other people at work
    1 Very Much
    2 Somewhat
    3 A Little
    4 Not At All
    5 Don't Have Any Such Person

12. Your spouse, friends and relatives
    1 Very Much
    2 Somewhat
    3 A Little
    4 Not At All
    5 Don't Have Any Such Person

WORK HAZARDS

FORM NUMBER: 02, FORM REVISION 01

STUDY ID:

Please answer each of the following questions as they apply to you.

1. Does your job primarily involve providing direct service to specific groups of people or client populations?
    1 = Yes
    2 = No

2. How often does your job expose you to verbal abuse and/or confrontations with clients or the general public?
    1 Never
    2 Occasionally
    3 Sometimes
    4 Fairly Often
    5 Very Often

3. How often does your job expose you to the threat of physical harm or injury?
    1 Never
    2 Occasionally
    3 Sometimes
    4 Fairly Often
    5 Very Often

4. How often have you been physically assaulted within the past 12 months while performing your job?
    1 Never
    2 Occasionally
    3 Sometimes
    4 Fairly Often
    5 Very Often

5. How often does your job personally subject you to potential legal liability?
    1 Never
    2 Occasionally
    3 Sometimes
    4 Fairly Often
    5 Very Often

WORK LIMITATIONS

FORM NUMBER: 22, FORM REVISION: 01

STUDY ID:

We are interested in learning about any work disability you may have had in the last 12 months. Please answer the following questions.

1. Within the past 12 months, has the kind or amount of work you can do been limited by any disability?
    1 = Yes
    2 = No

If you answered No, skip the remaining questions on this form.

2. Was this disability caused by your job?
    1 = Yes
    2 = No

If you answered No, skip to question 4, below.

3. Was this disability a result of an accident at work?
    1 = Yes
    2 = No

4. Have you applied for financial benefits as a result of this disability?
    1 = Yes
    2 = No

5. Do you currently receive financial benefits as a result of this disability?
    1 = Yes
    2 = No

WORKLOAD AND RESPONSIBILITY

FORM NUMBER: 10, FORM REVISION: 01

STUDY ID:

The next few items are concerned with various aspects of your work activities. Please indicate how much of each aspect you have on your job by writing a number in the box provided.

1. How much slowdown in the work load do you experience?
   1 Hardly Any
   2 A Little
   3 Some
   4 A Lot
   5 A Great Deal

2. How much time do you have to think and contemplate?
   1 Hardly Any
   2 A Little
   3 Some
   4 A Lot
   5 A Great Deal

3. How much work load do you have?
   1 Hardly Any
   2 A Little
   3 Some
   4 A Lot
   5 A Great Deal

4. What quantity of work do others expect you to do?
   1 Hardly Any
   2 A Little
   3 Some
   4 A Lot
   5 A Great Deal

5. How much time do you have to do all your work?
   1 Hardly Any
   2 A Little
   3 Some
   4 A Lot
   5 A Great Deal

6. How many projects, assignments, or tasks do you have?
   1 Hardly Any
   2 A Little
   3 Some
   4 A Lot
   5 A Great Deal

7. How many lulls between heavy work load periods do you have?
    1 Hardly Any
    2 A Little
    3 Some
    4 A Lot
    5 A Great Deal

8. How much responsibility do you have for the future of others?
    1 Hardly Any
    2 A Little
    3 Some
    4 A Lot
    5 A Great Deal

9. How much responsibility do you have for the job security of others?
    1 Hardly Any
    2 A Little
    3 Some
    4 A Lot
    5 A Great Deal

10. How much responsibility do you have for the morale of others?
    1 Hardly Any
    2 A Little
    3 Some
    4 A Lot
    5 A Great Deal

11. How much responsibility do you have for the welfare and lives of others?
    1 Hardly Any
    2 A Little
    3 Some
    4 A Lot
    5 A Great Deal

YOUR JOB

FORM NUMBER: 04, FORM REVISION: 01

STUDY ID:

How accurate are each of the following statements in describing your job?

1. I feel certain about how much authority I have.
    1 Very Inaccurate
    2 Mostly Inaccurate
    3 Slightly Inaccurate
    4 Uncertain
    5 Slightly Accurate
    6 Mostly Accurate
    7 Very Accurate

2. There are clear, planned goals and objectives for my job.
    1 Very Inaccurate
    2 Mostly Inaccurate
    3 Slightly Inaccurate
    4 Uncertain
    5 Slightly Accurate
    6 Mostly Accurate
    7 Very Accurate

3. I have to do things that should be done differently.
    1 Very Inaccurate
    2 Mostly Inaccurate
    3 Slightly Inaccurate
    4 Uncertain
    5 Slightly Accurate
    6 Mostly Accurate
    7 Very Accurate

4. I know that I have divided my time properly.
    1 Very Inaccurate
    2 Mostly Inaccurate
    3 Slightly Inaccurate
    4 Uncertain
    5 Slightly Accurate
    6 Mostly Accurate
    7 Very Accurate

5. I receive an assignment without the help I need to complete it.
    1 Very Inaccurate
    2 Mostly Inaccurate
    3 Slightly Inaccurate
    4 Uncertain
    5 Slightly Accurate
    6 Mostly Accurate
    7 Very Accurate

6. I know what my responsibilities are.
    1 Very Inaccurate
    2 Mostly Inaccurate
    3 Slightly Inaccurate
    4 Uncertain
    5 Slightly Accurate
    6 Mostly Accurate
    7 Very Accurate

7. I have to bend or break a rule or policy in order to carry out an assignment.
    1 Very Inaccurate
    2 Mostly Inaccurate
    3 Slightly Inaccurate
    4 Uncertain
    5 Slightly Accurate
    6 Mostly Accurate
    7 Very Accurate

8. I work with two or more groups who operate quite differently.
    1 Very Inaccurate
    2 Mostly Inaccurate
    3 Slightly Inaccurate
    4 Uncertain
    5 Slightly Accurate
    6 Mostly Accurate
    7 Very Accurate

9. I know exactly what is expected of me.
    1 Very Inaccurate
    2 Mostly Inaccurate
    3 Slightly Inaccurate
    4 Uncertain
    5 Slightly Accurate
    6 Mostly Accurate
    7 Very Accurate

10. I receive incompatible requests from two or more people.
    1 Very Inaccurate
    2 Mostly Inaccurate
    3 Slightly Inaccurate
    4 Uncertain
    5 Slightly Accurate
    6 Mostly Accurate
    7 Very Accurate

11. I do things that are apt to be accepted by one person and not accepted by others.
    1 Very Inaccurate
    2 Mostly Inaccurate
    3 Slightly Inaccurate
    4 Uncertain
    5 Slightly Accurate
    6 Mostly Accurate
    7 Very Accurate

12. I receive an assignment without adequate resources and materials to execute it.
    1 Very Inaccurate
    2 Mostly Inaccurate
    3 Slightly Inaccurate
    4 Uncertain
    5 Slightly Accurate
    6 Mostly Accurate
    7 Very Accurate

13. Explanation is clear about what has to be done on my job.
    1 Very Inaccurate
    2 Mostly Inaccurate
    3 Slightly Inaccurate
    4 Uncertain
    5 Slightly Accurate
    6 Mostly Accurate
    7 Very Accurate

14. I work on unnecessary things.
    1 Very Inaccurate
    2 Mostly Inaccurate
    3 Slightly Inaccurate
    4 Uncertain
    5 Slightly Accurate
    6 Mostly Accurate
    7 Very Accurate

YOUR JOB FUTURE

FORM NUMBER: 21, FORM REVISION: 01

STUDY ID:

In the future, some jobs will be changing while others will be staying the same. Here are some questions which deal with this topic.

1. How certain are you about what your future career picture looks like?
    1 Somewhat Uncertain
    2 A Little Uncertain
    3 Somewhat Certain
    4 Fairly Certain
    5 Very Certain

2. How certain are you of the opportunities for promotion and advancement which will exist in the next few years?
    1 Somewhat Uncertain
    2 A Little Uncertain
    3 Somewhat Certain
    4 Fairly Certain
    5 Very Certain

3. How certain are you about whether your job skills will be of use and value five years from now?
    1 Somewhat Uncertain
    2 A Little Uncertain
    3 Somewhat Certain
    4 Fairly Certain
    5 Very Certain

4. How certain are you about what your responsibilities will be six months from now?
    1 Somewhat Uncertain
    2 A Little Uncertain
    3 Somewhat Certain
    4 Fairly Certain
    5 Very Certain

5. If you lost your job, how certain are you that you could support yourself?
    1 Somewhat Uncertain
    2 A Little Uncertain
    3 Somewhat Certain
    4 Fairly Certain
    5 Very Certain
