# Supplementary material for: Gender differences in the relationships between perceived individual-level occupational stress and hazardous alcohol consumption among Japanese teachers: A cross-sectional study
Source: PLoS One. 2018 Sep 20;13(9):e0204248. doi: 10.1371/journal.pone.0204248 (PMC6147498; doi:10.1371/journal.pone.0204248)
Supplement: S3 File — (PDF) [file pone.0204248.s003.pdf]

SCORING KEY FOR NIOSH GENERIC JOB STRESS QUESTIONNAIRE

1. Physical Environment Evaluation: Page 2, Questions 1 to 10.

Compute average of items, reverse score 1, 2, 5, 9, 10.

2. Role Conflict: Pages 2 and 3, Questions 3, 5, 7, 8, 10, 11, 12, 14.

Compute average of items, no reverse scoring.

Alpha = 0.82 (From data from 700 Newfoundland NURSES)

3. Role Ambiguity: Pages 2 and 3, Questions 1, 2, 4, 6, 9, 13.

Compute average of items, reverse score all items.7

Alpha = 0.74 (NURSES)

4. Intragoup Conflict: Page 3, Questions 1 to 8.

Compute average of items, reverse score 1, 5, 7, 8.

Alpha = 0.86 (NURSES)

5. Intergroup Conflict: Pages 3 and 4, Questions 9 to 16.

Compute average of items, reverse score 10, 12, 14.

Alpha = 0.85 (NURSES)

NOTE: Principal Factor and Principal Components Analyses (using oblique rotation) of the NURSES responses to the Conflict Items indicate that there are three factors present:

Intragroup Conflict (Questions 2, 3, 4, 6, and 15)

Alpha = 0.79

Intergroup Conflict (Questions 9, 10, 11, 12, 13, 14, and 16)

Alpha = 0.85

Group Cohesion (Questions 1, 5, 7, and 8)

Alpha = 0.81

6. Job Future Ambiguity: Page 4, Questions 1 to 4.

Compute average of items, reverse score all items.

Alpha = 0.65 (NURSES)

7. Perceived Control: Pages 5 and 6, Questions 1 to 16.

Compute average of items, no reverse scoring.

Alpha = 0.90 (NURSES)

NOTE: Principal Factor Analysis (oblique rotation) with Screen test indicates the presence of four factors:

Task Control (Questions 1, 3, 4, 5, 6, 15, and 16)

Alpha = 0.85

Decision Control (Questions 8, 10, 11, and 13)

Alpha = 0.74

Physical Environment Control (Questions 7 and 14)

Alpha = 0.79

Resource Control (Questions 2 and 12)

Alpha = 0.82

8. Lack of Alternative Opportunities: Page 6, Questions 1 to 3.

Compute average of items, no reverse scoring.

Alpha = 0.80

9. Social Support from Supervisor: Pages 6 and 7, Questions 1A, 2A, 3A, 4A.

Compute average of items, no reverse scoring.

Alpha = 0.88 (0.87 from NIOSH study of 6000 postal workers)

10. Social Support from Co-workers: Pages 6 and 7, Questions 1B, 2B, 3B, 4B.

Compute average of items, no reverse scoring.

Alpha = 0.84 (0.85 Postal Workers)

11. Social Support from Family/Friends: Pages 6 and 7, Questions 1C, 2C, 3C, 4C.

Compute average of items, no reverse scoring.

Alpha = 0.85 (0.76 Postal Workers)

12. Quantitative Workload: Page 7, Questions 1 to 4, Page 8, Questions 1 to 7.

Compute average of items, reverse score Page 8, Questions 1, 2, 5, 7.

Alpha = 0.85 (NURSES)

13. Variance in Workload: Page 7 and top of Page 8, Questions 5 to 7.

Compute average of items, no reverse scoring.

Alpha = 0.86 (NURSES)

NOTE: Principal Factors and Principal Components Analyses (using oblique rotation) of responses to all workload items by Nurses indicated slightly different factors:

Quantitative Workload (Page 8, Questions 1, 2, 3, 4, 5, 7).

Alpha = 0.75

Variance in Workload (Page 7 and top of Page 8, Questions 1 to 7)

Alpha = 0.90

14. Responsibility for People: Bottom page 8, Questions 8 to 11.

Compute average of items, no reverse scoring.

Alpha = 0.62 (NURSES)

15. Skill Underutilization: Top Page 8, Questions 8 to 10.

Compute average of items, reverse score all items.

Alpha = 0.73 (NURSES)

16. Mental Demands: Page 9, Questions 1 to 5.

Compute average of items, reverse score 1, 2, 3.

Alpha = 0.75 (0.71 Postal Workers)

17. Non-Work Activities: Page 9, Questions 1 to 7.

Compute sum of items, scoring "yes" as 1 and "no" as 0.

Alpha = 0.

18. Type A Personality: Bottom Pages 14 and 15, Questions 1 to 20.

Computer average of items reverse score Questions 3, 6, 8, 9, 11, 12, 14, 15, 16, 18.

Alpha = 0.85 (Postal Workers)

19. Self-Esteem: Bottom of Page 9 and top of Page 10, Questions 1 to 10.

Compute average of items, reverse score Questions 2, 3, 6, 7, 9.

Alpha = 0.85 (NURSES)

20. Somatic Complaints: Page 10, Questions 1 to 17.

Compute average of items, no reverse scoring.

Alpha = 0.87 (NURSES)

21. Job Satisfaction: Top page 14, Questions 1 to 4.

Compute average of items reverse score all items.

Alpha = 0.83 (NURSES)

NIOSH GENERIC JOB STRESS QUESTIONNAIRE - GENERAL JOB INFORMATION

1. How long have you worked for your present employer?
 Years
 Months

2. What is your current job title?

3. How long have you worked in this job?
 Years
 Months

4. Please circle the most appropriate description of your situation:
 1 Full-time permanent employee
 2 Full-time temporary employee
 3 Part-time permanent employee
 4 Casual
 5 Other (specify)

5. Circle the description that comes closest to your present work shift:
 1 Rotating eight-hour shift
 2 Rotating twelve-hour shift
 3 Permanent day shift
 4 Permanent evening shift
 5 Permanent night shift
 6 Other (specify)

6. How long have you worked the shift you circled above?
 Years
 Months

7. If you work on a rotating shift, what rotation pattern do you follow?

Eight-hour shift:
1 Day to evening to night
2 Night to evening to day
3 No set pattern
Twelve-hour shift:
4 day to night
5 night to day
6 No set pattern

8. How many times a week do you change shifts?
1 [0] I don't change
2 2-times
3 More than 2-times
4 Other (specify)

9. How many hours do you normally work per week in your job (not counting overtime)?
hours

10. How many hours overtime do you work in your job in an average week?
hours

11. How many hours per week do you work on any other job?
hours (Please mark "0" if no other job)

PHYSICAL ENVIRONMENT

Please indicate whether the following statements about your job are True or False. Circle 1 for True and 2 for False per item.

1. The level of noise in the area(s) in which I work is usually high.
1 True
2 False

2. The level of lighting in the area(s) in which I work is usually poor.
1 True
2 False

3. The temperature of my work area(s) during the summer is usually comfortable.
1 True
2 False

4. The temperature of my work area(s) during the winter is usually comfortable.
1 True
2 False

5. The humidity in my work area(s) is usually either too high or too low.
1 True
2 False

6. The level of air circulation in my work area(s) is good.
   1 True
   2 False

7. The air in my work area(s) is clean and free of pollution.
   1 True
   2 False

8. In my job, I am well protected from exposure to dangerous substances (e.g. radiation, medications, and anesthetic gases, etc.).
   1 True
   2 False

9. The overall quality of the physical environment where I work is poor.
   1 True
   2 False

10. My work area(s) is/are awfully crowded.
   1 True
   2 False

YOUR JOB

How accurate are each of the following statements in describing your job? Use the response categories as 1 = Very Inaccurate, 2 = Mostly Inaccurate, 3 = Slightly Inaccurate, 4 = Uncertain, 5 = Slightly Accurate, 6 = Mostly Accurate, 7 = Very Accurate.

1. I feel certain about how much authority I have.
   1 Very Inaccurate
   2 Mostly Inaccurate
   3 Slightly Inaccurate
   4 Uncertain
   5 Slightly Accurate
   6 Mostly Accurate
   7 Very Accurate

2. There are clear, planned goals and objectives for my job.
   1 Very Inaccurate
   2 Mostly Inaccurate
   3 Slightly Inaccurate
   4 Uncertain
   5 Slightly Accurate
   6 Mostly Accurate
   7 Very Accurate

3. I have to do things that should be done differently.

1 Very Inaccurate
2 Mostly Inaccurate
3 Slightly Inaccurate
4 Uncertain
5 Slightly Accurate
6 Mostly Accurate
7 Very Accurate

4. I know that I have divided my time properly.
    1 Very Inaccurate
    2 Mostly Inaccurate
    3 Slightly Inaccurate
    4 Uncertain
    5 Slightly Accurate
    6 Mostly Accurate
    7 Very Accurate

5. I receive an assignment without the help I need to complete it.
    1 Very Inaccurate
    2 Mostly Inaccurate
    3 Slightly Inaccurate
    4 Uncertain
    5 Slightly Accurate
    6 Mostly Accurate
    7 Very Accurate

6. I know what my responsibilities are.
    1 Very Inaccurate
    2 Mostly Inaccurate
    3 Slightly Inaccurate
    4 Uncertain
    5 Slightly Accurate
    6 Mostly Accurate
    7 Very Accurate

7. I have to bend or break a rule or policy in order to carry out an assignment.
    1 Very Inaccurate
    2 Mostly Inaccurate
    3 Slightly Inaccurate
    4 Uncertain
    5 Slightly Accurate
    6 Mostly Accurate
    7 Very Accurate

8. I work with two or more groups who operate quite differently.
    1 Very Inaccurate
    2 Mostly Inaccurate
    3 Slightly Inaccurate
    4 Uncertain

5 Slightly Accurate
6 Mostly Accurate
7 Very Accurate

9. I know exactly what is expected of me.
   1 Very Inaccurate
   2 Mostly Inaccurate
   3 Slightly Inaccurate
   4 Uncertain
   5 Slightly Accurate
   6 Mostly Accurate
   7 Very Accurate

10. I receive incompatible requests from two or more people.
    1 Very Inaccurate
    2 Mostly Inaccurate
    3 Slightly Inaccurate
    4 Uncertain
    5 Slightly Accurate
    6 Mostly Accurate
    7 Very Accurate

11. I do things that are apt to be accepted by one person and not accepted by others.
    1 Very Inaccurate
    2 Mostly Inaccurate
    3 Slightly Inaccurate
    4 Uncertain
    5 Slightly Accurate
    6 Mostly Accurate
    7 Very Accurate

12. I receive an assignment without adequate resources and materials to execute it.
    1 Very Inaccurate
    2 Mostly Inaccurate
    3 Slightly Inaccurate
    4 Uncertain
    5 Slightly Accurate
    6 Mostly Accurate
    7 Very Accurate

13. Explanation is clear about what has to be done on my job.
    1 Very Inaccurate
    2 Mostly Inaccurate
    3 Slightly Inaccurate
    4 Uncertain
    5 Slightly Accurate
    6 Mostly Accurate
    7 Very Accurate

14. I work on unnecessary things.
 1 Very Inaccurate
 2 Mostly Inaccurate
 3 Slightly Inaccurate
 4 Uncertain
 5 Slightly Accurate
 6 Mostly Accurate
 7 Very Accurate

CONFLICT AT WORK

Please answer the following questions about your work situation. Using the response categories 1 = Strongly Disagree, 2 = Moderately Disagree, 3 = Neither Agree nor Disagree, 4 = Moderately Agree, 5 = Strongly Agree

1. There is harmony within my group.
 1 Strongly Disagree
 2 Moderately Disagree
 3 Neither Agree nor Disagree
 4 Moderately Agree
 5 Strongly Agree

2. In our group, we have lots of bickering over who should do what job.
 1 Strongly Disagree
 2 Moderately Disagree
 3 Neither Agree nor Disagree
 4 Moderately Agree
 5 Strongly Agree

3. There is difference of opinion among the members of my group.
 1 Strongly Disagree
 2 Moderately Disagree
 3 Neither Agree nor Disagree
 4 Moderately Agree
 5 Strongly Agree

4. There is dissension in my group.
 1 Strongly Disagree
 2 Moderately Disagree
 3 Neither Agree nor Disagree
 4 Moderately Agree
 5 Strongly Agree

5. The members of my group are supportive of each other's ideas.
 1 Strongly Disagree
 2 Moderately Disagree
 3 Neither Agree nor Disagree
 4 Moderately Agree
 5 Strongly Agree

6. There are clashes between subgroups within my group.
    1 Strongly Disagree
    2 Moderately Disagree
    3 Neither Agree nor Disagree
    4 Moderately Agree
    5 Strongly Agree

7. There is friendliness among the members of my group.
    1 Strongly Disagree
    2 Moderately Disagree
    3 Neither Agree nor Disagree
    4 Moderately Agree
    5 Strongly Agree

8. There is "we" feeling among members of my group.
    1 Strongly Disagree
    2 Moderately Disagree
    3 Neither Agree nor Disagree
    4 Moderately Agree
    5 Strongly Agree

9. There are disputes between my group and other groups.
    1 Strongly Disagree
    2 Moderately Disagree
    3 Neither Agree nor Disagree
    4 Moderately Agree
    5 Strongly Agree

10. There is agreement between my group and other groups.
    1 Strongly Disagree
    2 Moderately Disagree
    3 Neither Agree nor Disagree
    4 Moderately Agree
    5 Strongly Agree

11. Other groups withhold information necessary for the attainment of our group tasks.
    1 Strongly Disagree
    2 Moderately Disagree
    3 Neither Agree nor Disagree
    4 Moderately Agree
    5 Strongly Agree

12. The relationship between my group and other groups is harmonious in attaining the overall organizational goals.
    1 Strongly Disagree
    2 Moderately Disagree
    3 Neither Agree nor Disagree
    4 Moderately Agree
    5 Strongly Agree

13. There is lack of mutual assistance between my group and other groups.
    1 Strongly Disagree
    2 Moderately Disagree
    3 Neither Agree nor Disagree
    4 Moderately Agree
    5 Strongly Agree

14. There is cooperation between my group and other groups.
    1 Strongly Disagree
    2 Moderately Disagree
    3 Neither Agree nor Disagree
    4 Moderately Agree
    5 Strongly Agree

15. There are personality clashes between my group and other groups.
    1 Strongly Disagree
    2 Moderately Disagree
    3 Neither Agree nor Disagree
    4 Moderately Agree
    5 Strongly Agree

16. Other groups create problems for my group.
    1 Strongly Disagree
    2 Moderately Disagree
    3 Neither Agree nor Disagree
    4 Moderately Agree
    5 Strongly Agree

YOUR JOB FUTURE

In the future, some jobs will be changing while others will be staying the same. Here are some questions which deal with this topic.

1. How certain are you about what your future career picture looks like?
    1 Somewhat Uncertain
    2 A Little Uncertain
    3 Somewhat Certain
    4 Fairly Certain
    5 Very Certain

2. How certain are you of the opportunities for promotion and advancement which will exist in the next few years?
    1 Somewhat Uncertain
    2 A Little Uncertain
    3 Somewhat Certain
    4 Fairly Certain
    5 Very Certain

3. How certain are you about whether your job skills will be of use and value five years from now?
   1 Somewhat Uncertain
   2 A Little Uncertain
   3 Somewhat Certain
   4 Fairly Certain
   5 Very Certain

4. How certain are you about what your responsibilities will be six months from now?
   1 Somewhat Uncertain
   2 A Little Uncertain
   3 Somewhat Certain
   4 Fairly Certain
   5 Very Certain

5. If you lost your job, how certain are you that you could support yourself?
   1 Somewhat Uncertain
   2 A Little Uncertain
   3 Somewhat Certain
   4 Fairly Certain
   5 Very Certain

CONTROL SCALE

The next series of questions asks how much influence you now have in each of several areas. By influence we mean the degree to which you control what is done by others at work and have freedom to determine what you do yourself at work.

1. How much influence do you have over the variety of tasks you perform?
   1 Very Little
   2 Little
   3 A Moderate Amount
   4 Much
   5 Very Much

2. How much influence do you have over the availability of supplies and equipment you need to do your work?
   1 Very Little
   2 Little
   3 A Moderate Amount
   4 Much
   5 Very Much

3. How much influence do you have over the order in which you perform tasks at work?
   1 Very Little
   2 Little
   3 A Moderate Amount
   4 Much
   5 Very Much

4. How much influence do you have over the amount of work you do?
   1 Very Little
   2 Little
   3 A Moderate Amount
   4 Much
   5 Very Much

5. How much influence do you have over the <u>pace</u> of your work, that is how fast or slow you work?
   1 Very Little
   2 Little
   3 A Moderate Amount
   4 Much
   5 Very Much

6. How much influence do you have over the quality of the work that you do?
   1 Very Little
   2 Little
   3 A Moderate Amount
   4 Much
   5 Very Much

7. How much influence do you have over the arrangement and decoration of your work area?
   1 Very Little
   2 Little
   3 A Moderate Amount
   4 Much
   5 Very Much

8. How much influence do you have over the decisions concerning which individuals in your work unit do which tasks?
   1 Very Little
   2 Little
   3 A Moderate Amount
   4 Much
   5 Very Much

9. How much influence do you have over the hours or schedule that you work?
   1 Very Little
   2 Little
   3 A Moderate Amount
   4 Much
   5 Very Much

10. How much influence do you have over the decisions as to when things will be done in your work unit?
   1 Very Little
   2 Little
   3 A Moderate Amount
   4 Much

5 Very Much

11. How much do you influence the policies, procedures, and performance in your unit?
    1 Very Little
    2 Little
    3 A Moderate Amount
    4 Much
    5 Very Much

12. How much influence do you have over the availability of materials you need to do your work?
    1 Very Little
    2 Little
    3 A Moderate Amount
    4 Much
    5 Very Much

13. How much influence do you have over the training of other workers in your unit?
    1 Very Little
    2 Little
    3 A Moderate Amount
    4 Much
    5 Very Much

14. How much influence do you have over the arrangement of furniture and other work equipment in your unit?
    1 Very Little
    2 Little
    3 A Moderate Amount
    4 Much
    5 Very Much

15. To what extent can you do your work ahead and take a short rest break during work hours?
    1 Very Little
    2 Little
    3 A Moderate Amount
    4 Much
    5 Very Much

16. In general, how much influence do you have over work and work-related factors?
    1 Very Little
    2 Little
    3 A Moderate Amount
    4 Much
    5 Very Much

EMPLOYMENT OPPORTUNITIES

The next four questions ask you to evaluate your feelings about your job in relationship to other jobs you might be able to get. Please respond to each item by placing a check mark next to the response that best indicates your feelings about the question.

1. How easy would it be for you to find a suitable job with another employer?
   - 1 Very Easy
   - 2 Quite Easy
   - 3 Fairly Easy
   - 4 Not Quite so Easy
   - 5 Not Easy at All

2. How easy would it be for you to find a job as good as the one you now have with another employer?
   - 1 Very Easy
   - 2 Quite Easy
   - 3 Fairly Easy
   - 4 Not Quite so Easy
   - 5 Not Easy at All

3. How would you describe the number of available jobs, with all types of employers, for a person with your qualifications?
   - 1 Very Easy
   - 2 Quite Easy
   - 3 Fairly Easy
   - 4 Not Quite so Easy
   - 5 Not Easy at All

4. How likely is it that you would have to move out of your local area to find a suitable job with another employer?
   - 1 Very Easy
   - 2 Quite Easy
   - 3 Fairly Easy
   - 4 Not Quite so Easy
   - 5 Not Easy at All

SOCIAL SUPPORT

1. How much does your immediate supervisor (boss) go out of his/her way to do things to make your work life easier for you?
   - 1 Don’t have any such person
   - 2 Not at all
   - 3 A Little
   - 4 Somewhat
   - 5 Very Much

2. How much do other people at work go out of their way to do things to make your work life easier for you?
   - 1 Don’t have any such person
   - 2 Not at all
   - 3 A Little

4 Somewhat
5 Very Much

3. How much do your spouse, friends, and relatives go out of their way to make your work life easier for you?
    1 Don't have any such person
    2 Not at all
    3 A Little
    4 Somewhat
    5 Very Much

4. How easy is it to talk with your immediate supervisor (boss)?
    1 Don't have any such person
    2 Not at all
    3 A Little
    4 Somewhat
    5 Very Much

5. How easy is it to talk to other people at work?
    1 Don't have any such person
    2 Not at all
    3 A Little
    4 Somewhat
    5 Very Much

6. How easy is it to talk with your spouse, friends, and relatives?
    1 Don't have any such person
    2 Not at all
    3 A Little
    4 Somewhat
    5 Very Much

7. How much can your immediate supervisor (boss) be relied on when things get tough at work?
    1 Don't have any such person
    2 Not at all
    3 A Little
    4 Somewhat
    5 Very Much

8. How much can other people at work are relied on when things get tough at work?
    1 Don't have any such person
    2 Not at all
    3 A Little
    4 Somewhat
    5 Very Much

9. How much can your spouse, friends, and relatives be relied on when things get tough at work?
    1 Don't have any such person
    2 Not at all
    3 A Little

4 Somewhat
5 Very Much

10. How much is your immediate supervisor (boss) willing to listen to your personal problems?
    1 Don't have any such person
    2 Not at all
    3 A Little
    4 Somewhat
    5 Very Much

11. How much are <u>other people at work willing to listen</u> to your personal problems?
    1 Don't have any such person
    2 Not at all
    3 A Little
    4 Somewhat
    5 Very Much

12. How much are your spouse, friends, and relatives willing to listen to your personal problems?
    1 Don't have any such person
    2 Not at all
    3 A Little
    4 Somewhat
    5 Very Much

JOB REQUIREMENTS

Now we would like you to indicate <u>how often</u> certain things happen at your job. Please write a number in the space next to each question.

1. How often does your job require you to work <u>very fast</u>?
    1 Rarely
    2 Occasionally
    3 Sometimes
    4 Fairly Often
    5 Very Often

2. How often does your job require you to work <u>very hard</u>?
    1 Rarely
    2 Occasionally
    3 Sometimes
    4 Fairly Often
    5 Very Often

3. How often does your job leave you with <u>little</u> time to get things done?
    1 Rarely
    2 Occasionally
    3 Sometimes
    4 Fairly Often
    5 Very Often

4. How often is there a great deal to be done?
    1 Rarely
    2 Occasionally
    3 Sometimes
    4 Fairly Often
    5 Very Often

5. How often is there a marked increase in the work load?
    1 Rarely
    2 Occasionally
    3 Sometimes
    4 Fairly Often
    5 Very Often

6. How often is there a marked increase in the amount of concentration required on your job?
    1 Rarely
    2 Occasionally
    3 Sometimes
    4 Fairly Often
    5 Very Often

7. How often is there a marked increase in how fast you have to think?
    1 Rarely
    2 Occasionally
    3 Sometimes
    4 Fairly Often
    5 Very Often

8. How often does your job let you use the skills and knowledge you learned in school?
    1 Rarely
    2 Occasionally
    3 Sometimes
    4 Fairly Often
    5 Very Often

9. How often are you given a chance to do the things you do best?
    1 Rarely
    2 Occasionally
    3 Sometimes
    4 Fairly Often
    5 Very Often

10. How often can you use the skills from your previous experience and training?
    1 Rarely
    2 Occasionally
    3 Sometimes
    4 Fairly Often
    5 Very Often

WORKLOAD AND RESPONSIBILITY

The next few items are concerned with various aspects of your work activities. Please indicate how much of each aspect you have on your job by writing a number in the space provided at the end of the question.

1. How much slowdown in the work load do you experience?
   1 Hardly Any
   2 A Little
   3 Some
   4 A Lot
   5 A Great Deal

2. How much time do you have to think and contemplate?
   1 Hardly Any
   2 A Little
   3 Some
   4 A Lot
   5 A Great Deal

3. How much work load do you have?
   1 Hardly Any
   2 A Little
   3 Some
   4 A Lot
   5 A Great Deal

4. What quantity of work do others expect you to do?
   1 Hardly Any
   2 A Little
   3 Some
   4 A Lot
   5 A Great Deal

5. How much time do you have to do all your work?
   1 Hardly Any
   2 A Little
   3 Some
   4 A Lot
   5 A Great Deal

6. How many projects, assignments, or tasks do you have?
   1 Hardly Any
   2 A Little
   3 Some
   4 A Lot
   5 A Great Deal

7. How many lulls between heavy work load periods do you have?
   1 Hardly Any

2 A Little
3 Some
4 A Lot
5 A Great Deal

8. How much responsibility do you have for the future of others?
   1 Hardly Any
   2 A Little
   3 Some
   4 A Lot
   5 A Great Deal

9. How much responsibility do you have for the job security of others?
   1 Hardly Any
   2 A Little
   3 Some
   4 A Lot
   5 A Great Deal

10. How much responsibility do you have for the morale of others?
   1 Hardly Any
   2 A Little
   3 Some
   4 A Lot
   5 A Great Deal

11. How much responsibility do you have for the welfare and lives of others?
   1 Hardly Any
   2 A Little
   3 Some
   4 A Lot
   5 A Great Deal

MENTAL DEMANDS

Please indicate the degree to which you agree or disagree with the following statements about your job by entering a number in the space provided at the end of each statement.

1. My job requires a great deal of concentration.
   1 Strongly Agree
   2 Slightly Agree
   3 Slightly Disagree
   4 Strongly Disagree

2. My job requires me to remember many different things.
   1 Strongly Agree
   2 Slightly Agree
   3 Slightly Disagree
   4 Strongly Disagree

3. I must keep my mind on my work at all times.
    1 Strongly Agree
    2 Slightly Agree
    3 Slightly Disagree
    4 Strongly Disagree

4. I can take it easy and still get my work done.
    1 Strongly Agree
    2 Slightly Agree
    3 Slightly Disagree
    4 Strongly Disagree

5. I can let my mind wander and still do the work.
    1 Strongly Agree
    2 Slightly Agree
    3 Slightly Disagree
    4 Strongly Disagree

NON-WORK ACTIVITIES

1. Do you work on another job in addition to this one?
    0 = No
    1 = Yes

2. Do you have children at home?
    0 = No
    1 = Yes

3. Do you have primary responsibility for child care duties?
    0 = No
    1 = Yes

4. Do you have primary responsibility for housecleaning duties?
    0 = No
    1 = Yes

5. Do you have primary responsibility for the care of an elderly or disabled person on a regular basis?
    0 = No
    1 = Yes

6. Are you going to school and taking courses for credit toward a degree?
    0 = No
    1 = Yes

7. Do you belong to a voluntary or religious organization at which you spend at least 5 to 10 hours per week?
    0 = No
    1 = Yes

HOW YOU FEEL ABOUT YOURSELF

1. On the whole, I am satisfied with myself.
   1 Strongly Disagree
   2 Disagree
   3 Neither Agree or Disagree
   4 Agree
   5 Strongly Agree

2. I feel I do not have much to be proud of.
   1 Strongly Disagree
   2 Disagree
   3 Neither Agree or Disagree
   4 Agree
   5 Strongly Agree

3. I certainly feel useless at times.
   1 Strongly Disagree
   2 Disagree
   3 Neither Agree or Disagree
   4 Agree
   5 Strongly Agree

4. I feel that I'm a person of worth, at least on an equal basis with others.
   1 Strongly Disagree
   2 Disagree
   3 Neither Agree or Disagree
   4 Agree
   5 Strongly Agree

5. I feel that I have a number of good qualities.
   1 Strongly Disagree
   2 Disagree
   3 Neither Agree or Disagree
   4 Agree
   5 Strongly Agree

6. All in all, I am inclined to feel that I am a failure.
   1 Strongly Disagree
   2 Disagree
   3 Neither Agree or Disagree
   4 Agree
   5 Strongly Agree

7. I wish I could have more respect for myself.
   1 Strongly Disagree
   2 Disagree
   3 Neither Agree or Disagree
   4 Agree

5 Strongly Agree

8. I am able to do things as well as most other people.
    1 Strongly Disagree
    2 Disagree
    3 Neither Agree or Disagree
    4 Agree
    5 Strongly Agree

9. At times I think I am no good at all.
    1 Strongly Disagree
    2 Disagree
    3 Neither Agree or Disagree
    4 Agree
    5 Strongly Agree

10. I take a positive attitude toward myself.
    1 Strongly Disagree
    2 Disagree
    3 Neither Agree or Disagree
    4 Agree
    5 Strongly Agree

GENERAL HEALTH

This portion of the questionnaire contains items that are related to general health. These items are not necessarily related to severe physical illness but are things that people experience in their day to day lives. Please think of how often you have experienced any of the following statements during the past month by entering a number in the space provided at the end of each statement.

1. Your face became hot when you were not in a hot room or exercising.
    1 Never
    2 Occasionally
    3 Sometimes
    4 Fairly Often
    5 Very Often

2. You perspired excessively when you were not in a hot room or exercising.
    1 Never
    2 Occasionally
    3 Sometimes
    4 Fairly Often
    5 Very Often

3. Your mouth became dry.
    1 Never
    2 Occasionally
    3 Sometimes

4 Fairly Often
5 Very Often

4. Your muscles felt tight and tense.
    1 Never
    2 Occasionally
    3 Sometimes
    4 Fairly Often
    5 Very Often

5. You were bothered by a headache.
    1 Never
    2 Occasionally
    3 Sometimes
    4 Fairly Often
    5 Very Often

6. You felt as if the blood were rushing to your head.
    1 Never
    2 Occasionally
    3 Sometimes
    4 Fairly Often
    5 Very Often

7. You felt a lump in your throat or a choked-up feeling.
    1 Never
    2 Occasionally
    3 Sometimes
    4 Fairly Often
    5 Very Often

8. Your hands trembled enough to bother you.
    1 Never
    2 Occasionally
    3 Sometimes
    4 Fairly Often
    5 Very Often

9. You were bothered by shortness of breath when you were not working hard or exercising.
    1 Never
    2 Occasionally
    3 Sometimes
    4 Fairly Often
    5 Very Often

10. You were bothered by your heart beating hard.
    1 Never
    2 Occasionally
    3 Sometimes

4 Fairly Often
5 Very Often

11. Your hands sweated so that you felt damp and clammy.
    1 Never
    2 Occasionally
    3 Sometimes
    4 Fairly Often
    5 Very Often

12. You had spells of dizziness.
    1 Never
    2 Occasionally
    3 Sometimes
    4 Fairly Often
    5 Very Often

13. You were bothered by having an upset stomach or stomach ache.
    1 Never
    2 Occasionally
    3 Sometimes
    4 Fairly Often
    5 Very Often

14. You were bothered by your heart beating.
    1 Never
    2 Occasionally
    3 Sometimes
    4 Fairly Often
    5 Very Often

15. You were in ill health which affected your work.
    1 Never
    2 Occasionally
    3 Sometimes
    4 Fairly Often
    5 Very Often

16. You had a loss of appetite.
    1 Never
    2 Occasionally
    3 Sometimes
    4 Fairly Often
    5 Very Often

17. You had trouble sleeping at night.
    1 Never
    2 Occasionally
    3 Sometimes

4 Fairly Often
5 Very Often

HEALTH CONDITIONS

Within the past twelve months, has a doctor ever treated you for, or told you that you had: Please enter 1 = No and 2 = Yes in the space provided at the end of each condition.

1. Diabetes
   1 = No
   2 = Yes

2. Cancer
   1 = No
   2 = Yes

3. Hernia or rupture
   1 = No
   2 = Yes

4. Tuberculosis
   1 = No
   2 = Yes

5. Asthma
   1 = No
   2 = Yes

6. “High” blood pressure
   1 = No
   2 = Yes

7. Heart disease
   1 = No
   2 = Yes

8. Arthritis
   1 = No
   2 = Yes

9. Epilepsy
   1 = No
   2 = Yes

10. Glaucoma
    1 = No
    2 = Yes

11. Paralysis, tremor, or shaking
    1 = No
    2 = Yes

12. Kidney or bladder trouble
    1 = No
    2 = Yes

13. Lung or breathing problems
    1 = No
    2 = Yes

14. Stroke
    1 = No
    2 = Yes

15. Anemia
    1 = No
    2 = Yes

16. Gall Bladder, liver, or pancreas trouble
    1 = No
    2 = Yes

17. Thyroid trouble or goiter
    1 = No
    2 = Yes

18. Insomnia
    1 = No
    2 = Yes

19. Gastritis
    1 = No
    2 = Yes

20. Colitis
    1 = No
    2 = Yes

21. Stomach ulcer
    1 = No
    2 = Yes

22. Alcoholism
    1 = No
    2 = Yes

23. Emotional problems
1 = No
2 = Yes

24. Back problems
1 = No
2 = Yes

OTHER HEALTH INFORMATION

1. On an average day, how many of each of the following do you smoke? (Mark “0” if you don’t smoke.)
a Cigarettes
b Cigars
c Pipefuls of tobacco

2. During the past 6 months have you had any on the job accidents?
1 = Yes
2 = No

3. During the past month about how many days of sick leave did you take?
Days (Please enter “0” if none.)

4. During the past week how often did you experience the following: Please enter the number in the space provided at the end of each statement.

I was bothered by things that usually don’t bother me.
0 = Rarely or none of the time (less than 1 day)
1 = Some of the time (1-2 days)
2 = Occasionally or a moderate amount of time (3-4 days)
3 = Most or all of the time (5-7 days)

I did not feel like eating; my appetite was poor.
0 = Rarely or none of the time (less than 1 day)
1 = Some of the time (1-2 days)
2 = Occasionally or a moderate amount of time (3-4 days)
3 = Most or all of the time (5-7 days)

I felt that I could not shake off the blues even with help from my family or friends.
0 = Rarely or none of the time (less than 1 day)
1 = Some of the time (1-2 days)
2 = Occasionally or a moderate amount of time (3-4 days)
3 = Most or all of the time (5-7 days)

I felt that I was just as good as other people.
0 = Rarely or none of the time (less than 1 day)
1 = Some of the time (1-2 days)
2 = Occasionally or a moderate amount of time (3-4 days)

3 = Most or all of the time (5-7 days)

I had trouble keeping my mind on what I was doing.
0 = Rarely or none of the time (less than 1 day)
1 = Some of the time (1-2 days)
2 = Occasionally or a moderate amount of time (3-4 days)
3 = Most or all of the time (5-7 days)

I felt depressed.
0 = Rarely or none of the time (less than 1 day)
1 = Some of the time (1-2 days)
2 = Occasionally or a moderate amount of time (3-4 days)
3 = Most or all of the time (5-7 days)

I felt that everything I did was an effort.
0 = Rarely or none of the time (less than 1 day)
1 = Some of the time (1-2 days)
2 = Occasionally or a moderate amount of time (3-4 days)
3 = Most or all of the time (5-7 days)

I felt hopeful about the future.
0 = Rarely or none of the time (less than 1 day)
1 = Some of the time (1-2 days)
2 = Occasionally or a moderate amount of time (3-4 days)
3 = Most or all of the time (5-7 days)

I thought my life had been a failure.
0 = Rarely or none of the time (less than 1 day)
1 = Some of the time (1-2 days)
2 = Occasionally or a moderate amount of time (3-4 days)
3 = Most or all of the time (5-7 days)

I felt fearful.
0 = Rarely or none of the time (less than 1 day)
1 = Some of the time (1-2 days)
2 = Occasionally or a moderate amount of time (3-4 days)
3 = Most or all of the time (5-7 days)

My sleep was restless.
0 = Rarely or none of the time (less than 1 day)
1 = Some of the time (1-2 days)
2 = Occasionally or a moderate amount of time (3-4 days)
3 = Most or all of the time (5-7 days)

I was happy.
0 = Rarely or none of the time (less than 1 day)
1 = Some of the time (1-2 days)
2 = Occasionally or a moderate amount of time (3-4 days)
3 = Most or all of the time (5-7 days)

I talked less than usual.

0 = Rarely or none of the time (less than 1 day)
1 = Some of the time (1-2 days)
2 = Occasionally or a moderate amount of time (3-4 days)
3 = Most or all of the time (5-7 days)

I felt lonely.

0 = Rarely or none of the time (less than 1 day)
1 = Some of the time (1-2 days)
2 = Occasionally or a moderate amount of time (3-4 days)
3 = Most or all of the time (5-7 days)

People were unfriendly.

0 = Rarely or none of the time (less than 1 day)
1 = Some of the time (1-2 days)
2 = Occasionally or a moderate amount of time (3-4 days)
3 = Most or all of the time (5-7 days)

I enjoyed life.

0 = Rarely or none of the time (less than 1 day)
1 = Some of the time (1-2 days)
2 = Occasionally or a moderate amount of time (3-4 days)
3 = Most or all of the time (5-7 days)

I had crying spells.

0 = Rarely or none of the time (less than 1 day)
1 = Some of the time (1-2 days)
2 = Occasionally or a moderate amount of time (3-4 days)
3 = Most or all of the time (5-7 days)

I felt sad.

0 = Rarely or none of the time (less than 1 day)
1 = Some of the time (1-2 days)
2 = Occasionally or a moderate amount of time (3-4 days)
3 = Most or all of the time (5-7 days)

I felt that people disliked me.

0 = Rarely or none of the time (less than 1 day)
1 = Some of the time (1-2 days)
2 = Occasionally or a moderate amount of time (3-4 days)
3 = Most or all of the time (5-7 days)

I could not get “going”.

0 = Rarely or none of the time (less than 1 day)
1 = Some of the time (1-2 days)
2 = Occasionally or a moderate amount of time (3-4 days)
3 = Most or all of the time (5-7 days)

5. <u>During the past month</u> have you experienced any of the following: Please enter 1 = No or 2 = Yes in the slot at the end of each statement.

1 Cold or flu symptoms
1 = No
2 = Yes

2 Hay fever or allergy symptoms
1 = No
2 = Yes

3 Allergic skin rash
1 = No
2 = Yes

4 Slow healing wounds
1 = No
2 = Yes

5 Cold sores or fever blisters
1 = No
2 = Yes

6 Arthritis symptoms (swollen or painful joints)
1 = No
2 = Yes

7 Other illness (please explain)
1 = No
2 = Yes

BACKGROUND INFORMATION

1. What is your sex?
Female
Male

2. How old were you on your last birthday?
(Please enter age in the space provided at the end of the question.)

3. What is your marital status?
(Please enter the appropriate response in the space provided at the end of the question.)

4. If you have children living at home, how many are in each of the following age groups?
Children under 4 years
Children 4 through 12 years
Children 13 through 18 years
Children 19 and over

JOB SATISFACTION

We would like you to think about the <u>type of work you do in your job</u>. Please enter your answer on the space provided at the end of each question.

1. Knowing what you know now, if you had to decide all over again whether to take the type of job you now have, what would you decide?
    1 I would decide without hesitation to take the same job.
    2 I would have some second thoughts.
    3 I would decide definitely not to take this type of job.

2. If you were free right now to go into any type of job you wanted, what would your choice be?
    1 I would take the same job.
    2 I would take a different job.
    3 I would not want to work.

3. If a friend of yours told you he/she was interested in working in a job like yours, what would you tell him/her?
    1 I would strongly recommend it.
    2 I would have doubts about recommending it.
    3 I would advise against it.

4. All in all, how satisfied would you say you are with your job?
    1 I am very satisfied.
    2 I am somewhat satisfied.
    3 I am not too satisfied.
    4 I am not at all satisfied.

ACTIVITY LEVEL

Please indicate the degree to which each of the following statements is true or false as they apply to you. Please enter your answer on the space provided at the end of each statement.

1. I am more restless and fidgety than most people.
    1 Definitely False
    2 Mostly False
    3 Don't Know
    4 Mostly True
    5 Definitely True

2. I ordinarily work quickly and energetically.
    1 Definitely False
    2 Mostly False
    3 Don't Know
    4 Mostly True
    5 Definitely True

3. I am rather deliberate in telephone conversations.
    1 Definitely False
    2 Mostly False

3 Don’t Know
4 Mostly True
5 Definitely True

4. I am often in a hurry.
   1 Definitely False
   2 Mostly False
   3 Don’t Know
   4 Mostly True
   5 Definitely True

5. In conversation I often gesture with hands and head.
   1 Definitely False
   2 Mostly False
   3 Don’t Know
   4 Mostly True
   5 Definitely True

6. I rarely drive a car too fast.
   1 Definitely False
   2 Mostly False
   3 Don’t Know
   4 Mostly True
   5 Definitely True

7. As a boy or girl I preferred work in which I could move around.
   1 Definitely False
   2 Mostly False
   3 Don’t Know
   4 Mostly True
   5 Definitely True

8. People consider me to be rather quiet.
   1 Definitely False
   2 Mostly False
   3 Don’t Know
   4 Mostly True
   5 Definitely True

9. I usually speak more softly than most people.
   1 Definitely False
   2 Mostly False
   3 Don’t Know
   4 Mostly True
   5 Definitely True

10. My handwriting is rather fast.
    1 Definitely False
    2 Mostly False

3 Don’t Know
4 Mostly True
5 Definitely True

11. I often work slowly and leisurely.
    1 Definitely False
    2 Mostly False
    3 Don’t Know
    4 Mostly True
    5 Definitely True

12. I prefer to linger over a meal and enjoy it.
    1 Definitely False
    2 Mostly False
    3 Don’t Know
    4 Mostly True
    5 Definitely True

13. I like to drive a car rather fast when there is no speed limit.
    1 Definitely False
    2 Mostly False
    3 Don’t Know
    4 Mostly True
    5 Definitely True

14. I like work that is slow and deliberate.
    1 Definitely False
    2 Mostly False
    3 Don’t Know
    4 Mostly True
    5 Definitely True

15. I talk more slowly than most people.
    1 Definitely False
    2 Mostly False
    3 Don’t Know
    4 Mostly True
    5 Definitely True

16. I often let a problem work itself out by waiting.
    1 Definitely False
    2 Mostly False
    3 Don’t Know
    4 Mostly True
    5 Definitely True

17. I often try to persuade others to my point of view.
    1 Definitely False
    2 Mostly False

3 Don’t Know
4 Mostly True
5 Definitely True

18. I generally walk more slowly than most people.
1 Definitely False
2 Mostly False
3 Don’t Know
4 Mostly True
5 Definitely True

19. I eat rapidly even when there is plenty of time.
1 Definitely False
2 Mostly False
3 Don’t Know
4 Mostly True
5 Definitely True

20. I usually work quickly.
1 Definitely False
2 Mostly False
3 Don’t Know
4 Mostly True
5 Definitely True

PROBLEMS AT WORK

People deal with day to day problems at work in many ways. When faced with problems at work, how often do you do each of the following? Please enter a number in the space provided at the end of each statement.

1. Make a plan to solve the problem(s) and stick to it.
1 Rarely
2 Occasionally
3 Sometimes
4 Fairly Often
5 Very Often

2. Go on as if nothing has happened.
1 Rarely
2 Occasionally
3 Sometimes
4 Fairly Often
5 Very Often

3. Feel responsible for the problem(s).
1 Rarely
2 Occasionally
3 Sometimes
4 Fairly Often

5 Very Often

4. Daydream or wish that you could change the problem(s).
    1 Rarely
    2 Occasionally
    3 Sometimes
    4 Fairly Often
    5 Very Often

5. Talk to your boss or co-workers about the problem(s).
    1 Rarely
    2 Occasionally
    3 Sometimes
    4 Fairly Often
    5 Very Often

6. Become more involved in activities outside of work.
    1 Rarely
    2 Occasionally
    3 Sometimes
    4 Fairly Often
    5 Very Often
